# Supplementary material for: Analysis of targeted and whole genome sequencing of PacBio HiFi reads for a comprehensive genotyping of gene-proximal and phenotype-associated Variable Number Tandem Repeats
Source: PLoS Comput Biol. 2025 Apr 7;21(4):e1012885. doi: 10.1371/journal.pcbi.1012885 (PMC11975116; doi:10.1371/journal.pcbi.1012885)
Supplement: S1 Table — (PDF) [file pcbi.1012885.s001.pdf]

Supplementary

A. Supplementary

References for P-VNTRs are provided in the Supplementary Table S1.

Gene name	Phenotype and reference
ACAN	height[15,16], lumbar disc herniation[41]
PER3	age of onset for bipolar disorder[42,43]
PRDM9	recombination hotspot[15]
MUC7	bronchial asthma[44,45]
WDR7	amyotrophic lateral sclerosis (ALS)[46]
PLIN4	skeletal muscle disease[47]
IL1RN	Stroke, Atherosclerosis and Coronary Artery Disease (CAD)[48]
TMCO1	Glaucoma[17]
CUL4A	Decreased mean corpuscular hemoglobin[17]
IL4	Severe joint destruction in rheumatoid arthritis, polyarthritis (protective)[49]
MUC6	Alzhemier, late onset Alzheimer's disease (LOAD) and gene expression[50]
MUC6	Altered pTau pathology and AP2A2 gene expression (nearby)[50]
CACNA1C	Bipolar disorder and schizophrenia[14]
FXN	Friedreich ataxia (FA)[7]
NLRP3	Gastric Cancer[51]
EIF3H	Colorectal cancer and colon polyps[17]
NOS3	Renal Failure and gene expression[52–54]
GP1BA	ischemic stroke, coronary thrombosis, fatal myocardial infarction (MI)[55,56]
HCG22	Diffuse panbronchiolitis (DPB)[57]
MUC22	Diffuse panbronchiolitis (DPB)[57]
CNBP	Myotonic dystrophy type 2 (DM2)[58,59]
PRNP	Creutzfeldt-Jakob disease[60,61]
DRD4	ADHD, OCD[62–64]
ABCA7	Alzheimers[65]
C9orf72	ALS, frontotemporal dementia (FTD)[66]
MUC1	ADTKD[67]
NACA	atrial fibrillation[7,15]
SLC6A3	ADHD, Parkinson's disease, schizophrenia[68,69]
SLC6A3	ADHD, Parkinson's disease[7,70,71]
SLC6A3	higher inattention scores[70–72]
CEL	Monogenic diabetes and and Hereditary Pancreatitis[73]
NOP56	SCA36, a Type of Spinocerebellar Ataxia Accompanied by Motor Neuron Involvement[74]
HIC1	Metastatic Colorectal Cancer[75]
INS	T1D (type 1 diabetes) lower association: polycystic ovary syndrome, T2D, Obesity[76]
VWA1	motor neuropathy[77]

CSTB	autosomal recessive progressive myoclonus epilepsy (EPM1), Unverricht-Lundborg, Myoclonic Epilepsy Of Unverricht And Lundborg[78]
SLC6A4	ADHD, OCD, anxiety, SCZ[7,79,80]
SLC6A4	ADHD, Alzheimers disease, BPSD[7,80]
TCHH	Male pattern baldness score[7,16]
EIF4A3	Richieri-Costa-Pereira syndrome (RCPS)[81]
MAOA	bi-polar disorder, impulsivity and antisocial behaviour. Schizophrenia, Nichotine dependence[82,83]
TAF1	X-linked Dystonia-Parkinsonism (XDP)[84]
FRA16B	Fragile site FRA16B[85]
PCSK6	Degree of Handedness[86]
TENT5A	Height[16]
AVPR1A	Verbal learning and memory[87]
DUX4	Facioscapulohumeral muscular dystrophy (FSHD)[88,89]

Table S1. P-VNTR Genes, associated phenotypes and references.
