# Supplementary figures and images for: Analysis of targeted and whole genome sequencing of PacBio HiFi reads for a comprehensive genotyping of gene-proximal and phenotype-associated Variable Number Tandem Repeats
Source: PLoS Comput Biol. 2025 Apr 7;21(4):e1012885. doi: 10.1371/journal.pcbi.1012885 (PMC11975116; doi:10.1371/journal.pcbi.1012885)

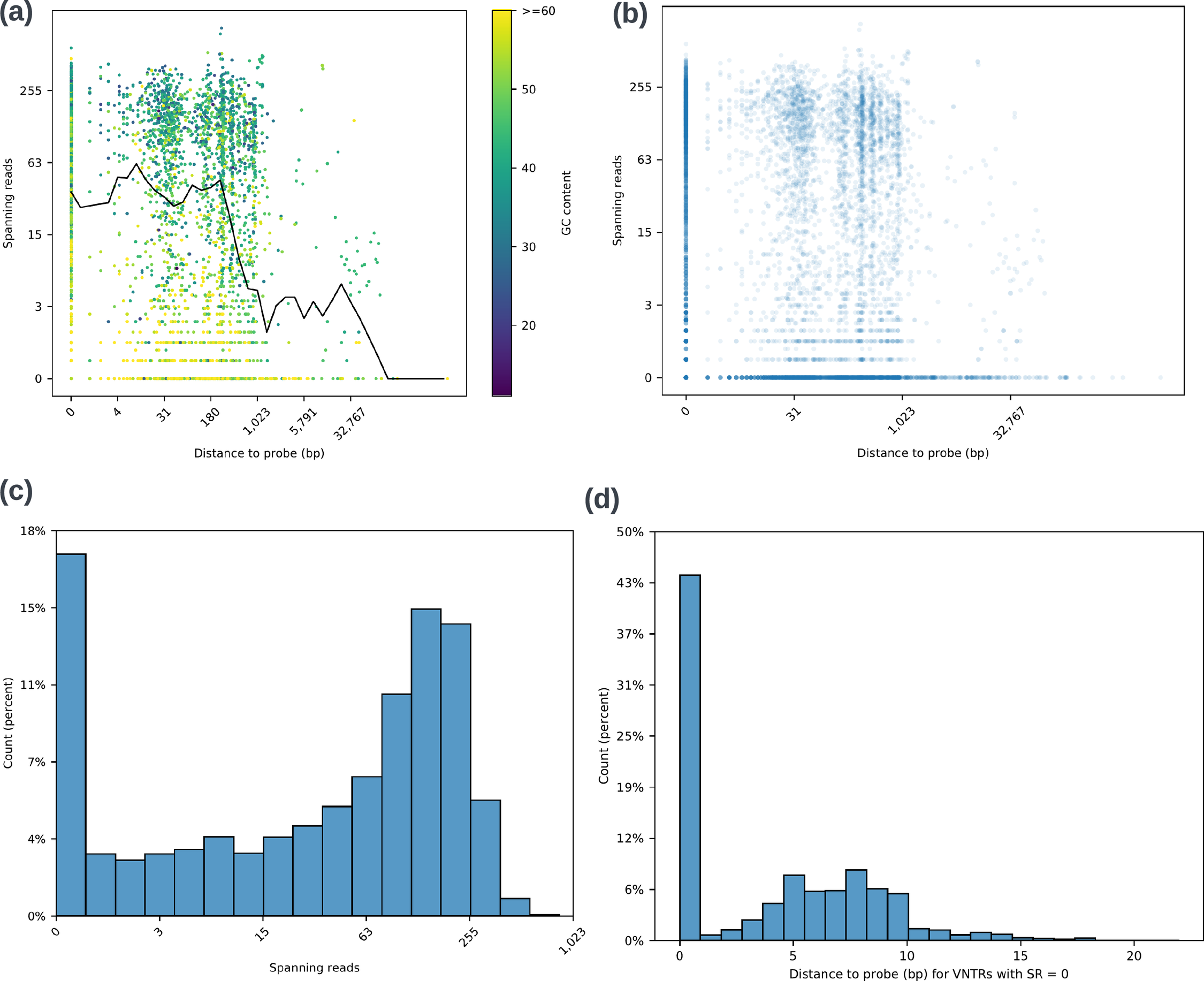

Supplement: S1 Fig — (a) The hue shows the GC content percentile for VNTR and 100 bp flanking regions. The black line represents the trendline computed as a rolling window, averaging the median of 5 consecutive columns. (b) the hue represents the density of overlapping points. (c) The histogram of spanning reads regardless of the distance to probe. (d) The histogram of the distance to probe for the VNTRs with zero spanning reads corresponding the horizontal line on zero spanning reads in (a) and (b) where overlapping points on the horizontal line hides the distance to probe distribution. (TIF) [file pcbi.1012885.s006.tif]

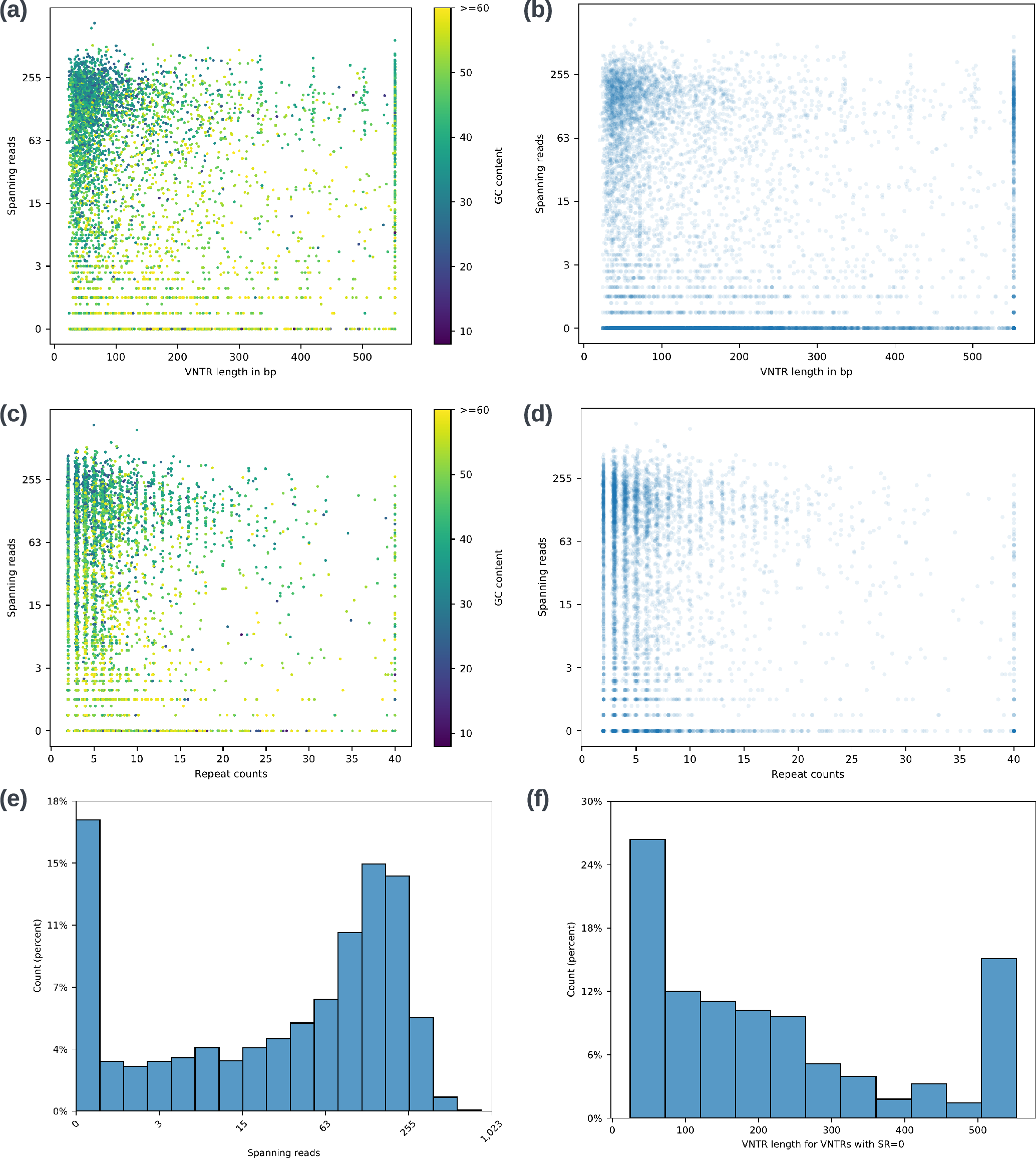

Supplement: S2 Fig — G-VNTRswith GC-content < 60% are plotted here. Spanning reads for each VNTR correspond to the median spanning reads across all samples (in log scale). (a) VNTR length up to 99th percentile shown on the X axis. Any VNTR length longer than that is projected to the 99th percentile value. The hue shows the GC content percentile for VNTR and 100 bp flanking regions. (b) The X axis is similar to (a). The hue shows the density of overlapping points. (c) The X axis indicates VNTR repeat counts instead of VNTR lengths. The hue shows the GC content percentile for VNTR and 100 bp flanking regions. (d) The hue shows the density of overlapping points. (e) The histogram of spanning reads regardless of the VNTR length. (f) The histogram of the VNTR length for the VNTRs with zero spanning reads corresponding the horizontal line on zero spanning reads in (a), (b), (c), and (d) where overlapping points on the horizontal line hides the VNTR length distribution. (TIF) [file pcbi.1012885.s007.tif]

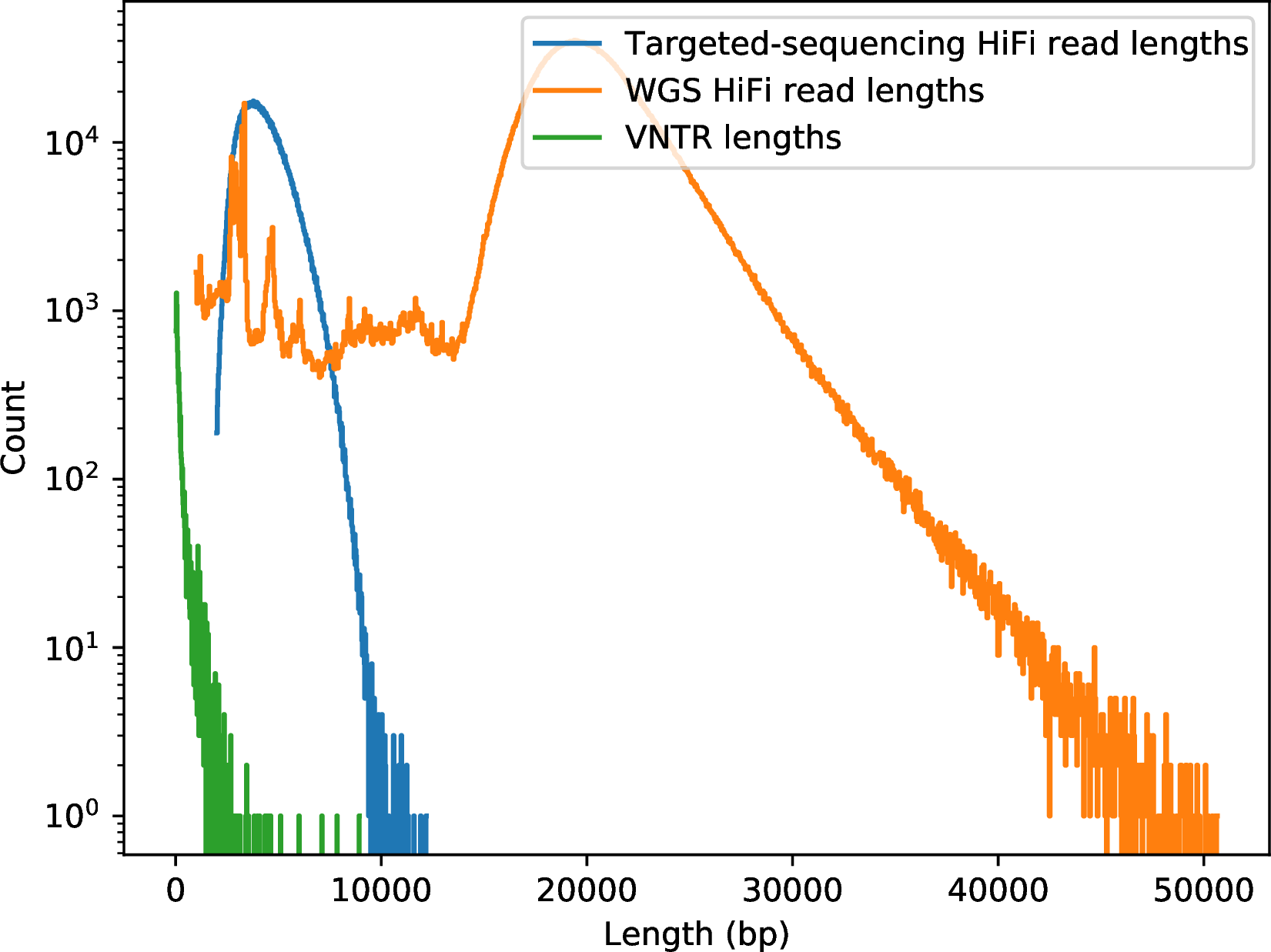

Supplement: S3 Fig — Read length distribution of a representative sample (id 7) from the targeted sequencing cohort is shown in blue. Similarly, the distribution for a representative sample from the whole genome sequencing cohort (id HG02559) is shown in orange. G-VNTR length distribution is shown with green color. The count in the Y axis is in log scale. In general, whole genome HiFi reads were significantly longer than targeted sequencing. However, both targeted sequencing and whole genome sequencing were long enough to span almost all G-VNTRs. (TIF) [file pcbi.1012885.s008.tif]

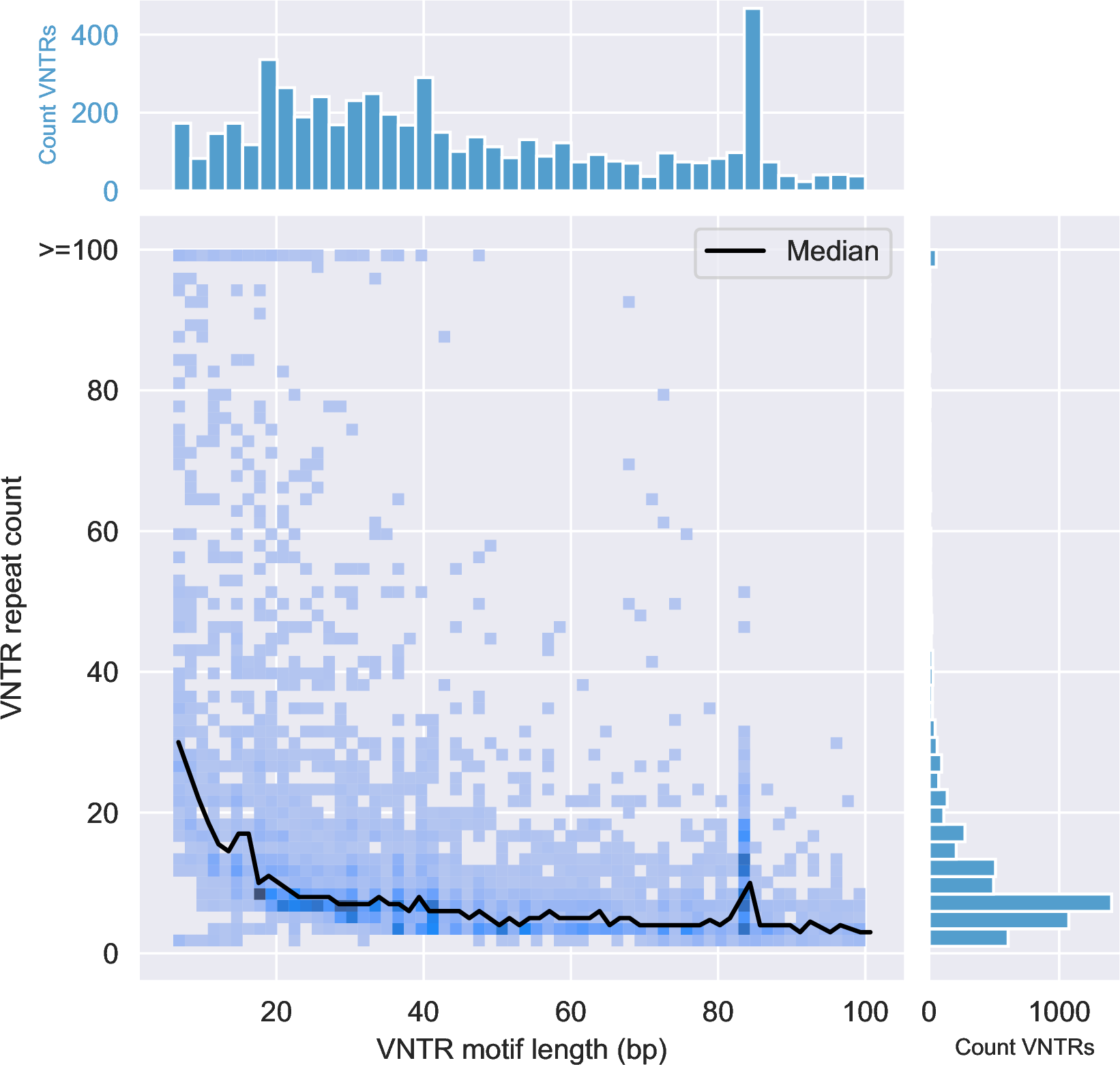

Supplement: S4 Fig — Each VNTR is represented by the mean repeat count across the alleles in the WGS cohort with darker squares representing higher density of VNTRs. The black trend line follows the mean value in each column. The top histogram presents the motif length regardless of motif counts. The right histogram shows VNTR alleles in terms of repeat counts based on the mean allele value in the WGS cohort. (TIF) [file pcbi.1012885.s009.tif]
