# Supplementary material for: Analysis of targeted and whole genome sequencing of PacBio HiFi reads for a comprehensive genotyping of gene-proximal and phenotype-associated Variable Number Tandem Repeats
Source: PLoS Comput Biol. 2025 Apr 7;21(4):e1012885. doi: 10.1371/journal.pcbi.1012885 (PMC11975116; doi:10.1371/journal.pcbi.1012885)
Supplement: Attachment — Submitted filename: Reviewer response letter.pdf [file pcbi.1012885.s010.pdf]

Cover letter

Dear PLOS Computational Biology Journal Editor,

We appreciate the time and effort that you and the reviewers have invested in reviewing our manuscript and providing constructive comments. We are pleased to submit our revised manuscript in response to the comments and suggestions provided by the reviewers as well as the detailed response to each of the reviewers' comments.

In this revision, we have addressed the reviewers' concerns by adding additional analyses, clarifying the methods section, expanded our introduction to cover a more extensive VNTR and targeted sequencing literature, and expanding the discussion on ONT reads, stutter error, and VNTRs in the segmental duplication regions.

In order to preserve the privacy of the participants providing samples for the targeted sequencing dataset, we renamed the sample ids in the revised manuscript, figures and the supplementary tables. The new ids are from 1 to 8 and assigned to the samples at a random order. We have uploaded the targeted sequencing Fastq files on the Sequence Read Archive (SRA) with Bioproject number PRJNA1209963. The samples are set to be released on February 20, 2025, but the release date can be moved earlier if the manuscript is published sooner. The submission information and samples are available right now through the reviewer link: https://dataview.ncbi.nlm.nih.gov/object/PRJNA1209963?reviewer=68lgek4bkk5ov6vfkk5bjglk5k

A few points to help make the reading experience of this response letter easier:

- To visually distinguish the reviewer comments from our responses, we kept the reviewer comments in **black** and marked all our responses in **blue**. Furthermore, to limit the need to switch between the response letter and the manuscript, if we changed the manuscript in response to a comment, we repeated the updates in this letter colored in **purple**.
- In the reviewer comments where reviewers quote a part of the manuscript, sometimes the line numbers in the manuscript are repeated in the middle of the sentence. Please have this in mind when reading the comments.

Thank you again for your consideration. We hope this revised manuscript meets the journal's standards and look forward to your feedback.

Sara Javadzadeh

Responses to Reviewers - 100% (75/75) of comments answered

Reviewer #1 - 0/11 comments left

The authors present a comparison of a new targeted HiFi sequencing approach to HiFi WGS for VNTRs. Although targeting can provide higher coverage of some targets with much less total reads, they find that most pathogenic VNTRs were not covered due to high GC and/or difficulty designing probes near the VNTR. I think this is a valuable comparison for the community and just have some suggestions to clarify.

1. The first time I read the abstract, I was confused whether the 3rd paragraph was talking about the targeted or WGS approach, so would be useful to start it saying "with the targeted approach"

Response. Changes applied exactly as requested.

2. "Specifically, these gene proximal ('G-VNTRs') lay within coding regions, 59 untranslated regions, or up to 500 bp upstream of the transcription start site (TSS), including promoter 60 and other regulatory regions. " Does this include introns?

Response. We have clarified this better in the revised manuscript as follows:

- Specifically, these gene proximal ('G-VNTRs') lay within the gene body (introns and exons), in untranslated regions, or up to 500 bp upstream of the transcription start site (TSS), including promoters and other regulatory regions.

3. "The enriched 79 DNA was sequenced using PacBio HiFi, with a median number of 1.5M long-reads per sample with 80 N50=4,207 bp " This is much shorter than HiFi WGS, so I think this is important enough to highlight and perhaps even mention in the abstract

Response. The reviewer makes a good point about the difference in the length of the HiFi reads in WGS and targeted sequencing. However, this is reasonable for our protocols. As described in the Methods section, "Genomic DNA extraction and fragmentation" paragraph, we sheared DNA to the target size of 6kbp that was suitable for our set of P-VNTRs and G-VNTRs which are typically less than 1 kbp in size."

To fully address the reviewer questions, we plotted the distributions of target VNTR lengths as well as the read lengths of the targeted and whole genome sequencing reads.

Figure R1 shows a histogram with the target G-VNTR length (in GRCh38 reference genome) with the maximum length of 8938 bp and 95 and 99 percentile of 1341 bp and 2373 bp respectively. Figure R1 is a modified version of Figure 1b from the manuscript, where the cumulative histogram was changed to a non-cumulative histogram specifically for this response. It is not included in the revised manuscript.

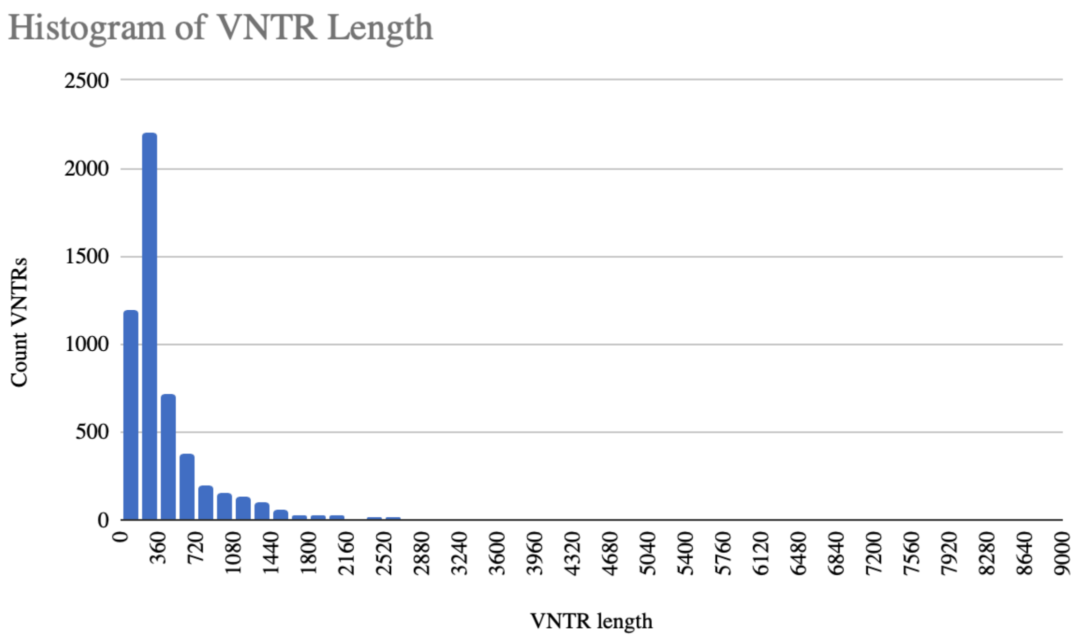

Figure R1. Reference length of targeted VNTRs

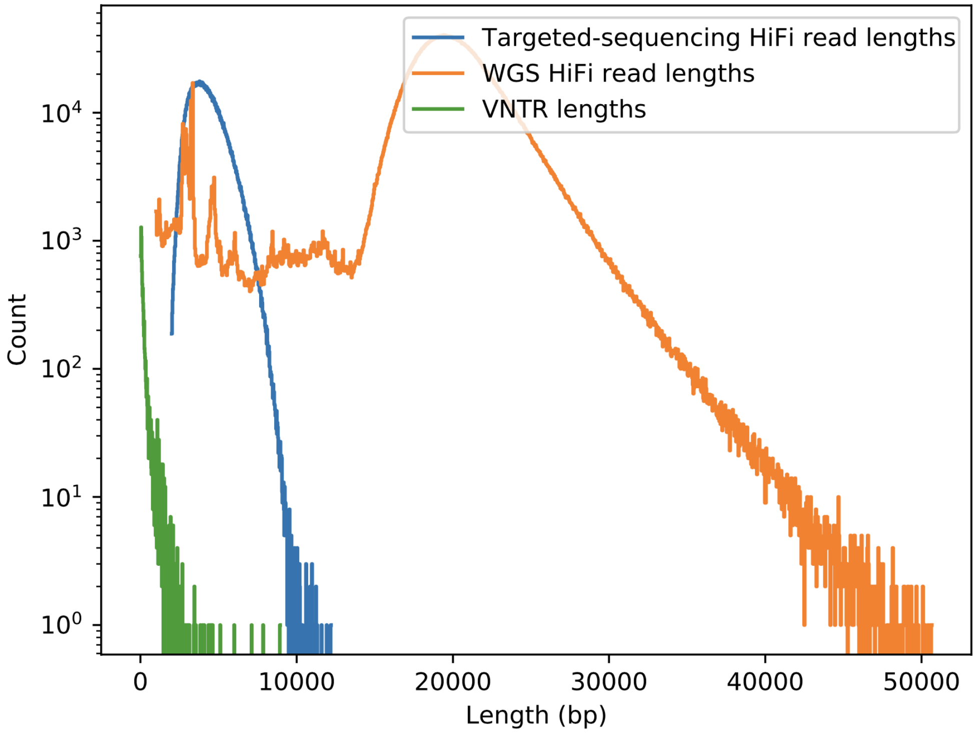

Figure R2, also Figure S3 in the revised manuscript.

- We observed that the read lengths with Median of 4.2 kbp (averaged among samples) is well above the 99 percentile of targeted VNTR lengths (2373 bp).
- The caption of new Figure S3 is as follows, "Read length distribution of a representative sample (id 7) from the targeted sequencing cohort is shown in blue. Similarly, the distribution for a representative sample from the whole genome sequencing cohort (id HG02559) is shown in orange. G-VNTR length distribution is shown with green color. The count in the Y axis is in log scale. In general, whole genome HiFi reads were significantly longer than targeted sequencing. However, both targeted sequencing and whole genome sequencing were long enough to span almost all G-VNTRs."
- We added the following to the manuscript:
    - We also tested, but did not find a correlation between the coverage and VNTR lengths … Notably, the targeted sequencing experiments were designed with the G-VNTR lengths in mind (See Methods). In the 8 samples, the median read length was in the range [4,027 − 4,429] bp (Supplementary Table S4). For comparison, the 95-th percentile of G-VNTR lengths was 1,341 (99 percentile: 2,373, max: 8,938; also see Supplementary Figure S3) which were easily spanned by the targeted reads. While the HiFi whole genome sequences were even longer, (median read length in range of [16 − 24] kbp), the targeted sequence length was sufficient to not impact coverage of G-VNTRs.

4. "The coverage was bi-modal in log scale with modes at 0 and 2^7 (Figure 2a) ". Maybe replace 2^7 with ~250

Response. Replacing 2^7 with 128 could cause confusion, as the distribution is bi-modal when spanning reads are shown on a log scale. Spanning reads in the range [65-128] are aggregated into a single bucket (2^7). Using 128 instead would inaccurately suggest a single peak at 128 spanning reads, rather than reflecting the aggregated range [65-128] forming a second peak. To avoid confusion, we removed the original sentence about the bi-modal distribution.

5. "Replicating genotype consistency analysis on the whole genome samples with Illumina and HiFi reads, 137 we observed a slightly lower genotype call consistency of 98.0%." I don't think this is significantly lower than the 98.01% for targeted, so would be better to say they are similar

Response. We thank the reviewer and have modified as requested.

6. "Additionally, we replicated the analysis on the Genome in a Bottle consortium29 and we observed 148 99.7% Mendelian consistency for the HG002, HG003 and HG004 trio using whole genome PacBio HiFi 149 reads, reflecting the robustness of genotyping accuracy using adVNTR on HiFi." Could the authors use the new TR benchmark from GIAB to evaluate

accuracy of targeted and WGS calls for HG002?
https://www.nature.com/articles/s41587-024-02225-z

Response. We agree with the reviewer about additional studies on Genome in a Bottle cohort. However, we do not have the targeted and WGS data available for the same samples. This means that we do not have targeted sequencing data for HG002 for the VNTR regions studied in this manuscript, and a direct comparison is not possible. We did do a direct comparison of genotypes when comparing Illumina and HiFi targeted sequencing, where we had data available for the same samples provided by COH.

We added the following in the introduction section to highlight the recent TR benchmark from GIAB as a valid resource.

- "Genome in a Bottle Consortium (GIAB)[18] has been an invaluable resource for tandem repeat analysis where English *et al* created a benchmark of tandem repeats based on the HG002 sample[19]."

7. "However, the length of the repeat unit itself (and therefore, the allele length) was 154 highly variable, suggesting that while polymerase slippage can cause changes in repeat counts, the range 155 of changes remains constrained." Could the authors explain more how they draw this conclusion?

Response. This is a good point. We did not have good evidence for that. Therefore we have kept the text observing that the length of the repeat unit was variable, but we removed the "suggesting that while …" and the rest of the sentence as it is unclear.

8. Related to the previous point, could the authors look at noise in the sequencing reads for targeted vs WGS to better understand whether PCR introduces more noise due to slippage?

Response. We did not have WGS and targeted sequencing data available for the same samples in this study. This limitation in data availability restricts our ability to accurately compare noise directly in WGS and targeted sequencing.

In the current literature, stutter error is generally observed for short tandem repeats (with shorter motifs) during PCR amplification. The error is more prominent as the repeat unit (motif) length decreases, and most pronounced when performing PCR for dinucleotide and mononucleotide repeats (Ellegren, 2004*). Loh *et al* calculated the stutter error in microsatellites (short tandem repeats or STRs) in experiments with targeted sequencing with hybridization and PCR (Loh, 2024*). In Figure 2C of their publication, they showed that stutter is predominant in STRs with motifs of length 2bp, present but less prevalent in STRs with motifs of length 3bp and minimal in STRs with motifs of length 4bp (Loh, 2024*). Therefore, as motif length increases, the stutter error decreases in the hybridization targeted approach with PCR. Here, we considered VNTRs with minimum motif length of 6bp, confirming that we do not expect stutter errors to be dominant in VNTRs.

We recognize that our study utilized targeted sequencing data that was not designed with a focus on computing stutter errors. It utilized data acquired for a breast cancer study. Specifically, the loci did not include male chrX TRs where a single allele is expected and all other lengths can be attributed to stutter error.

In the discussion, we have added the following text:

- “In this study, we did not compare differences in stutter errors between targeted and whole genome sequencing. Testing for stutters is typically performed on chromosome X of male samples, where any genotype other than the mode is likely to be a stutter error. Our targeted study samples were used in other studies with Illumina sequencing[38], and no chrX TRs were targeted. It is possible that the accuracy of genotyping targeted VNTRs could be impacted by stutter errors arising during PCR. Published reports on Short Tandem Repeats (STRs) with motif length <= 4, however, suggest that stutter errors have a strong negative correlation with motif length, and they are uncommon in motifs of length 4 [37]. In contrast, our study exclusively looked at VNTRs where the motif length was at least 6 bp. For longer motifs, stutter errors are unlikely to change motif counts, and AdVNTR is robust to small changes in motif sequences, because it uses underlying Hidden Markov Models (HMMs) allowing insertions and deletions within each motif.”

Also in the revision, we provided aligned read lengths and repeat counts for a few example VNTRs and the sample with id 7 in the targeted cohort in figures RS1 to RS4 below. To maintain conciseness, we are providing figures RS1 to RS4 here but have not added them to the revised manuscript. As seen in figures RS1 to RS4, the repeat counts inferred from the read lengths by AdVNTR have increased consistency when compared to the read lengths (cut to the VNTR boundary) for the same sample and VNTR. This is because the HMMs in AdVNTR designed to accurately match the motifs even with a slight difference in length (i.e. short indel in the motif).

Another observation is that as the motif length increases (motif lengths decrease from RS1 to RS4), the erroneous reads where the read length is uncommon (i.e. < 10%) decrease. This is in line with the findings in the literature referenced above.

*References:

- Ellegren H. Microsatellites: simple sequences with complex evolution. Nature reviews genetics. 2004 Jun 1;5(6):435-45.
- Loh CA, Shields DA, Schwing A, Evrony GD. High-fidelity, large-scale targeted profiling of microsatellites. Genome Research. 2024 Jul 1;34(7):1008-26.

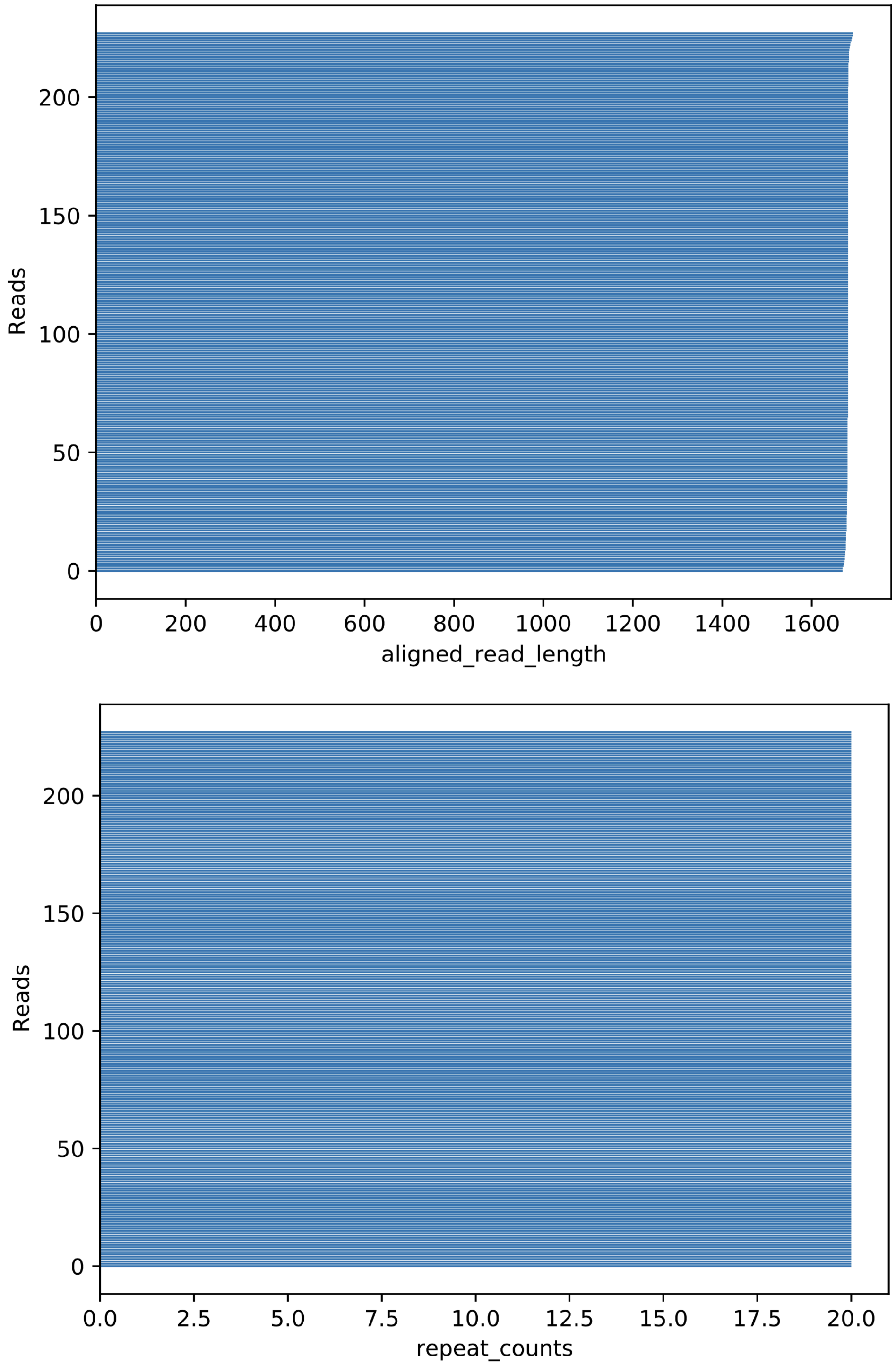

- Supplementary figure RS1. Read length and repeat counts plot for sample id 7 in the targeted sequencing cohort. Showing all the recruited spanning reads for VNTR with motif length 84 bp and GRCh38 reference length of 552 bp. **Top:** The reads are cut to the VNTR boundary. **Bottom:** Repeat counts are inferred using AdVNTR.

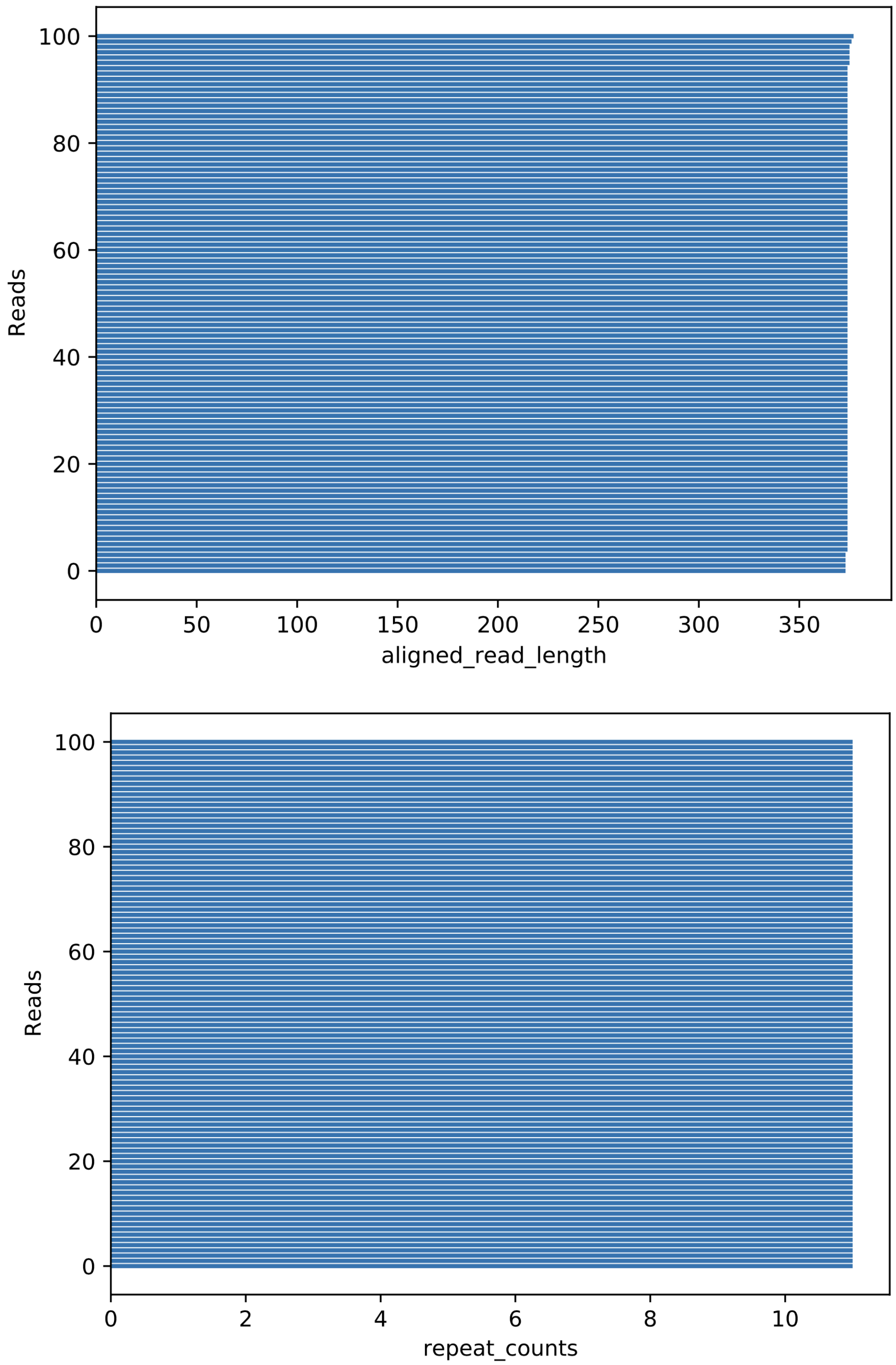

- Supplementary figure RS2. Read length and repeat counts plot for sample id 7 in the targeted sequencing cohort. Showing all the recruited spanning reads for VNTR with motif length 34 bp and GRCh38 reference length of 374 bp. **Top:** The reads are cut to the VNTR boundary. **Bottom:** Repeat counts are inferred using AdVNTR.

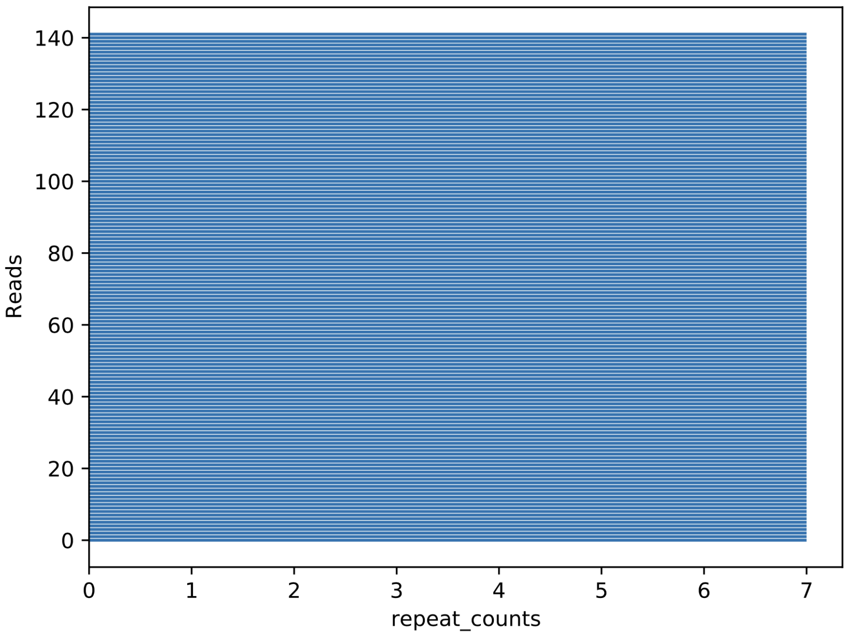

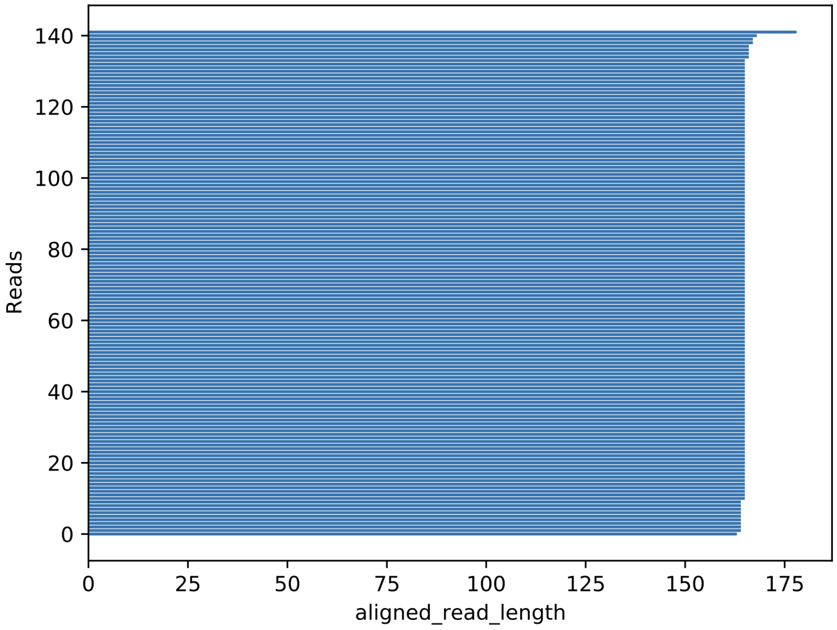

- Supplementary figure RS3. Read length and repeat counts plot for sample id 7 in the targeted sequencing cohort. Showing all the recruited spanning reads for VNTR with motif length 24 bp and GRCh38 reference length of 165 bp. **Top:** The reads are cut to the VNTR boundary. **Bottom:** Repeat counts are inferred using AdVNTR.

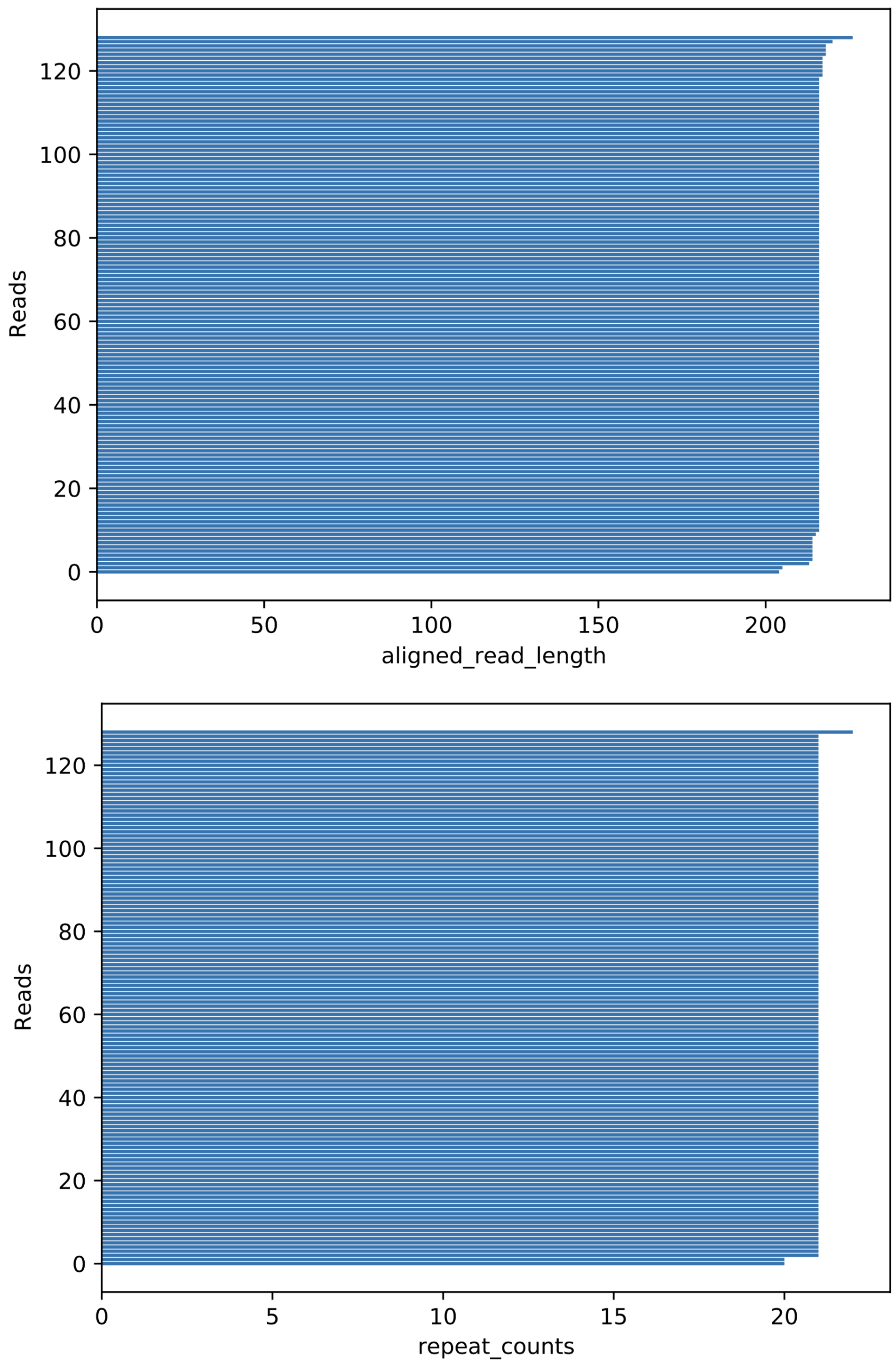

- Supplementary figure RS4. Read length and repeat counts plot for sample id 7 in the targeted sequencing cohort. Showing all the recruited spanning reads for VNTR with motif length 10 bp and GRCh38 reference length of 216 bp. **Top:** The reads are cut to the VNTR boundary. **Bottom:** Repeat counts are inferred using AdVNTR.

9. The link to G-VNTRs on github did not work in the pdf

Response. We provided the full link in the manuscript pdf and specified the exact section to find the database:

- The database of G-VNTRs and P-VNTRs is available online at the AdVNTR Github directory: https://github.com/mehrdadbakhtiari/adVNTR under "Data Requirements and Pre-trained Models (Databases)".

10. I noticed the LPA VNTR was missing from the P-VNTR list. Were very long VNTRs excluded?

Response. The reviewer points out LPA VNTR which has been associated with Lipoprotein(a) concentration in Mukamel *et al* (https://pmc.ncbi.nlm.nih.gov/articles/PMC8549062/). Based on their findings, this VNTR spans two exons of the LPA gene, its motif length is 5600 bp and its repeat count ranges from 2 to 40 (within the European population). This is too long for our VNTR list as the full VNTR length would fall beyond the HiFi read length of around 10 kbp and therefore, we would not have spanning reads for this VNTR for either targeted or WGS data. For this particular VNTR, methods such as the method used by Mukamel *et al* which is based on read depth and not dependent on spanning reads are most suitable.

11. I could not find a data availability statement for the targeted sequencing

Response. We have uploaded the targeted sequencing Fastq files on the Sequence Read Archive (SRA) with Bioproject number PRJNA1209963 and scheduled to be released on February 20, 2025 with the reviewer link: https://dataview.ncbi.nlm.nih.gov/object/PRJNA1209963?reviewer=68lgek4bkk5ov6vfkk5bjglk5k

- Data availability statement is updated to include the information on targeted sequencing data:
    - Targeted sequencing data generated in this study is available on Sequence Read Archive (SRA) with Bioproject number PRJNA1209963.

Reviewer #2 - 0/18 comments left

I was excited to read this paper, as HiFi targeting approaches for tandem repeats would be incredibly useful and are currently relatively difficult to scale to large numbers of loci. Overall it was an interesting and thorough analysis. Most of my comments are relatively minor.

Response. We thank the reviewer for their comment.

Major comments:

Can you predict which VNTRs can be targeted? This could help future researchers decide if they should attempt this approach.

Response. This is a very good point. To address this comment, we built linear and non-linear models to predict the expected number of spanning reads based on a number of features such as VNTR length, GC content, VNTR motif length, and distance to the designed probes. We did an 80% train and 20% test split on the G-VNTRs. We defined the problem both as regression (predict the number of spanning reads) and classification (binary, predict if there are at least 15 spanning reads). We worked with linear regression model, random forest regression model (non-linear), and linear and non-linear SGD classification models. Regression models had r^2 of 0.3 in the test dataset which is low. The classification models performed slightly better with accuracy in the range of 0.6-0.8 in the test dataset. Still, the prediction model is not accurate enough to predict the number of spanning reads (or if the number of spanning reads are above 15) for a given VNTR.

With our models, the feature importance of GC-content was within the range 0.3-0.4 dominating all the other features. The next most important feature was vntr-length with feature_importance of 0.2. This is in line with our findings on the manuscript about GC-content being the most important feature determining the number of spanning reads in VNTRs.

It is likely that there could be features that are not captured and could help with the accuracy of the models and building an accurate model is best to be left for a future study. We have not added these regression results to save space, but have added the following text to the discussion:

- "An interesting question that arises from our experiments is if we can predict in advance which VNTRs are amenable to targeted analysis. We attempted to address this question using regression methods on a number of features such as VNTR length, GC content, VNTR motif length, and distance to the designed probes. Unfortunately, the data were too sparse to yield insights. With additional data, the ability to identify VNTRs amenable to targeted sequencing would be very useful. Notably, our study identified over 4,000 VNTRs that can be utilized in targeted studies. While our approach focused on VNTRs with clinical relevance, a contrasting approach was followed in a related publication[36], where they focused on the largest set of microsatellites and deployed a number of filters to enrich microsatellites that are amenable to targeted sequencing."

Could adjustments to probe design help improve targeted sequencing? If so, what would you recommend? Why was a distance of 1000bp significant do you think?

Response. Based on our analysis, the factor contributing the most to the lack of spanning reads were the GC content of the VNTR. The regions with GC-content > 60% are difficult to amplify in the PCR step. It is likely that the designed probes could successfully capture the targeted DNA fragment, but amplification was unsuccessful, leaving us with zero spanning reads. For this reason, we believe that GC-content performs a larger part in the number of spanning reads rather than the designed probes.

We observed a significant drop of the median spanning reads after the distance of 1000 bp. Therefore, this threshold is data-oriented.

Did you observe evidence of allelic dropout in this study? If so, at what depth is this a problem?

Response. The reviewer points out the important concern of allelic dropout with regard to target enrichment. To our knowledge, allelic dropout has not been an issue with target hybridization methods, in contrast to amplicon-based approaches. Gray *et al* demonstrated that probe-based target enrichment with probes that are 120 bp long are not typically susceptible to allelic dropout as mismatches (up to seven) does not hinder hybridization (Gray 2015*). As the designed probes for our experiments are 120 bp and much longer than the PCR-primers (20bp), we do not believe that allelic dropout would be a concern for this study.

We added this in the introduction of the manuscript as well:

- Another advantage of probe-based target enrichment compared to the amplicon-based approaches is that allelic dropout is not a concern for probe-based enrichment. Gray *et al* demonstrated that target enrichment with probes that are 120 bp long are not typically susceptible to allelic dropout as mismatches (up to seven) does not hinder hybridization[26].
- *Reference: Gray PN, Dunlop CL, Elliott AM. Not all next generation sequencing diagnostics are created equal: understanding the nuances of solid tumor assay design for somatic mutation detection. Cancers. 2015 Jul 17;7(3):1313-32.

In section 2.2, specify the fragment size distributions pre- and post-target enrichment and comment on how this impacted read length. Were read lengths (and likely fragment lengths) shorter in the targeted data? Why? How much impact did this have on the number of spanning reads? Was there a particular allele size at which the read length was typically insufficient? This comment is particularly informed by ref 18, which describes using hybridization for HiFi sequencing. Their HiFi read length averaged at 6287 bp (5909–6819 bp, SD = 285 bp). These reads are much shorter than the typical 15kb of WGS. Is this the main reason for few spanning reads? Does this suggest it could work for more modest-sized VNTR loci?

Response. These are all good points raised by the reviewer. We would like to mention that our target enrichment experiment was designed for a predefined set of VNTRs (G-VNTRs and

P-VNTRs). Figure R1 shows a histogram of target G-VNTR lengths (in GRCh38 reference genome) with the maximum length of 8938 bp and 95 and 99 percentile of 1341 bp and 2373 bp respectively. A target fragment size of 6 kbp was chosen based on the length of target VNTRs. Figure R1 is a modified version of Figure 1b from the manuscript, where the cumulative histogram was changed to a non-cumulative histogram specifically for this response. It is not included in the revised manuscript.

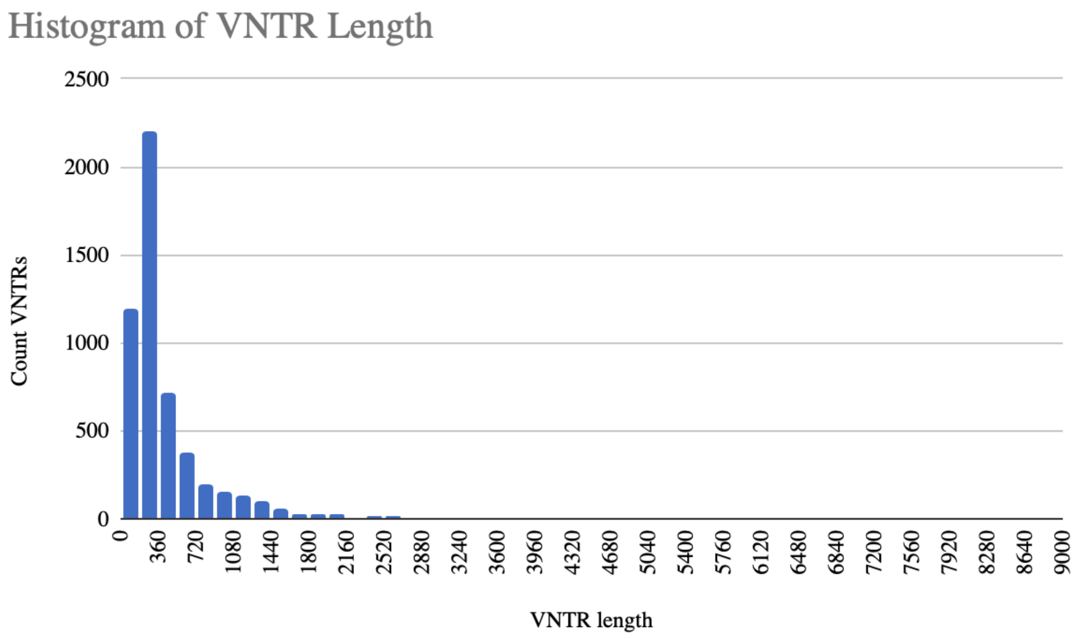

Figure R1. Reference length of targeted VNTRs

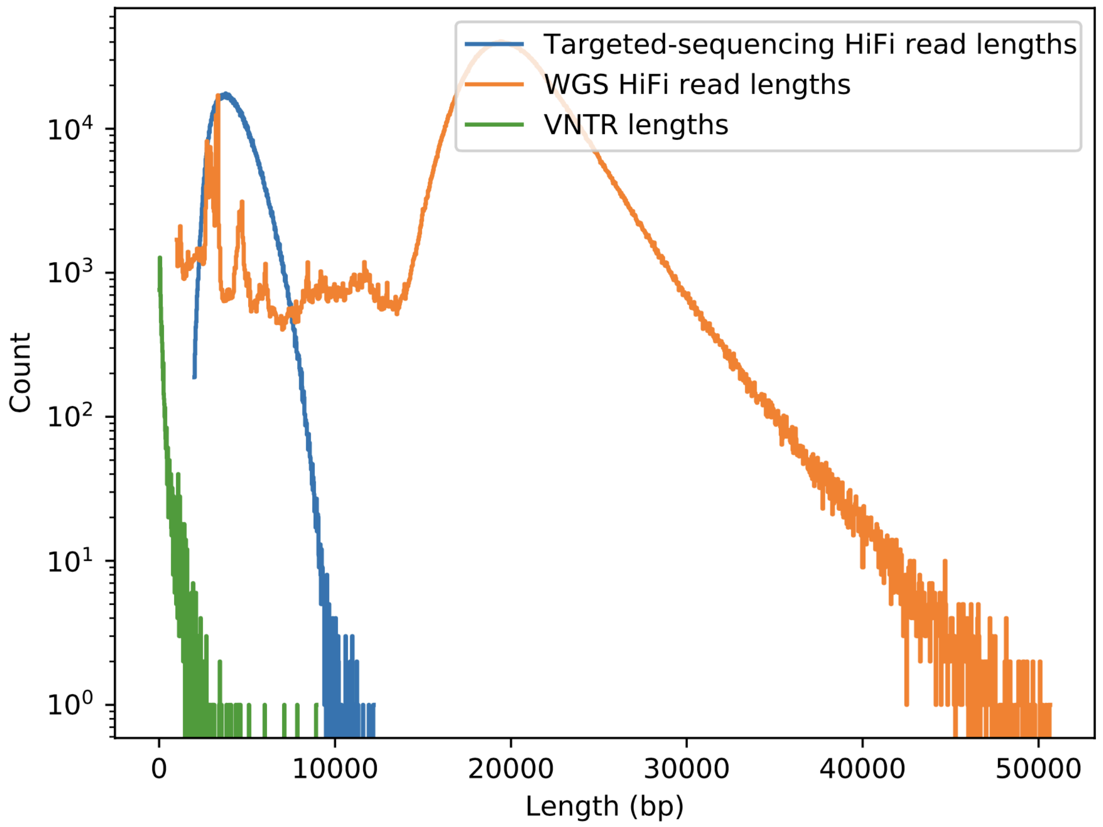

Figure R2, also Figure S3 in the revised manuscript.

- We observed that the read lengths with Median of 4.2 kbp (averaged among samples) is well above the 99 percentile of targeted VNTR lengths (2373 bp).
- The caption of new Figure S3 is as follows, "Read length distribution of a representative sample (id 7) from the targeted sequencing cohort is shown in blue. Similarly, the distribution for a representative sample from the whole genome sequencing cohort (id HG02559) is shown in orange. G-VNTR length distribution is shown with green color. The count in the Y axis is in log scale. In general, whole genome HiFi reads were significantly longer than targeted sequencing. However, both targeted sequencing and whole genome sequencing were long enough to span almost all G-VNTRs."
- To answer the follow up questions by the reviewer, comparing the target VNTR length histogram in Figure R1 with the reasoning above, VNTR allele length does not seem to be the main concern for spanning reads. Figure S2 in the manuscript (below) shows VNTRs with 0 spanning reads for a range of VNTR lengths even as small as 250bp. We highlighted that GC-content has a more prominent effect on spanning reads (as explained in Section 2.2 of the manuscript). This also suggests that "modest-sized VNTRs" (length not specified by the reviewer) will not necessarily get higher spanning reads.
- We added the following to the manuscript:
    - We also tested, but did not find a correlation between the coverage and VNTR lengths … Notably, the targeted sequencing experiments were designed with the

G-VNTR lengths in mind (See Methods). In the 8 samples, the median read length was in the range [4,027 − 4,429] bp (Supplementary Table S4). For comparison, the 95-th percentile of G-VNTR lengths was 1,341 (99 percentile: 2,373, max: 8,938; also see Supplementary Figure S3) which were easily spanned by the targeted reads. While the HiFi whole genome sequences were even longer, (median read length in range of [16 − 24] kbp), the targeted sequence length was sufficient to not impact coverage of G-VNTRs.

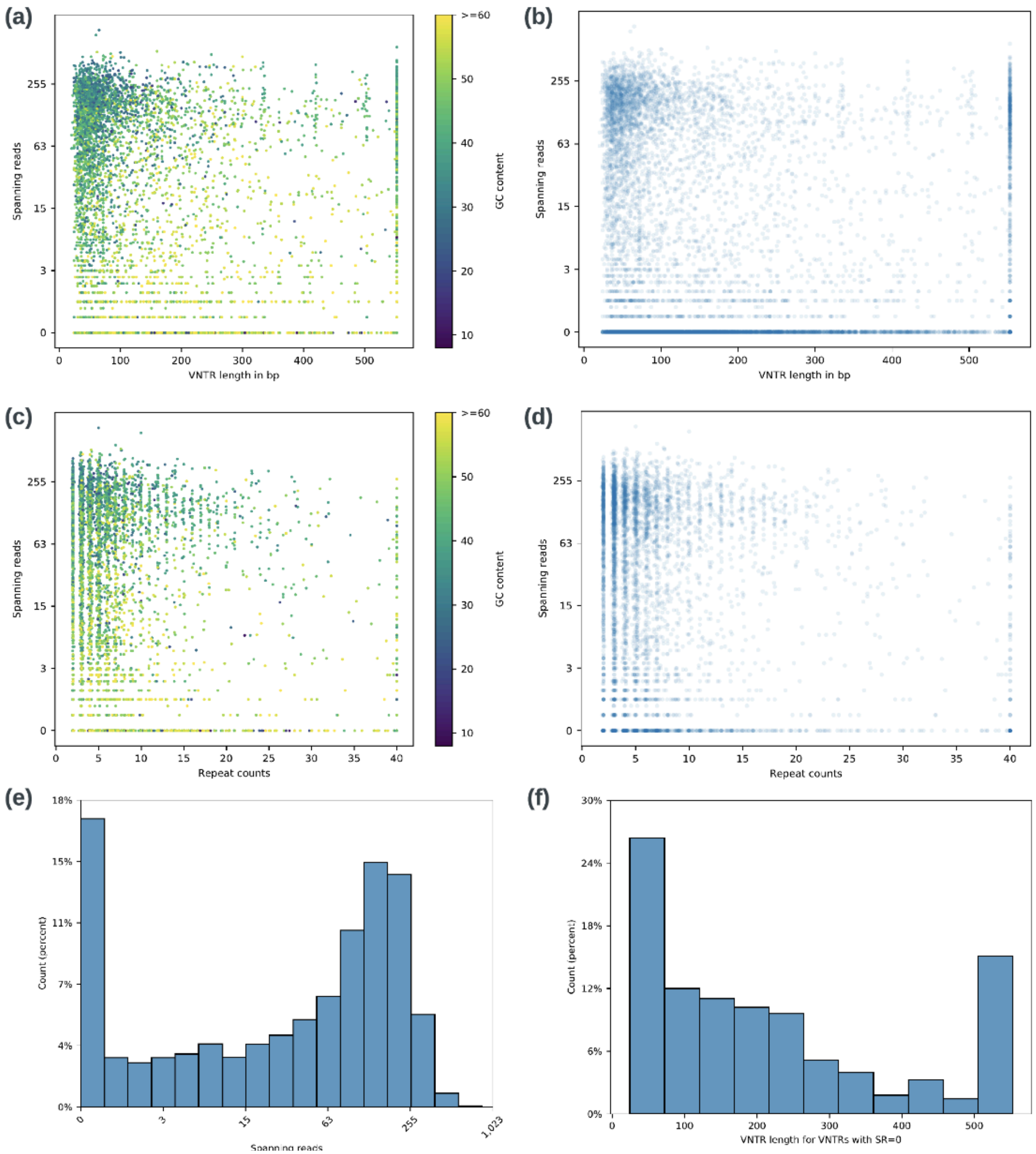

- Revised Figure S2 in the manuscript

"The coverage of G-VNTRs is tightly distributed on ≤ 20 repeat counts."
I struggled to understand this paragraph, and couldn't match it up with the supporting figure. So I'm not entirely sure what point it is making. I suggest editing for clarity.

Response. We agree that it was not clear. We have updated the manuscript to the following sentence:

- G-VNTR repeat counts decrease with increased motif length

Could you please elaborate in the results/discussion how much impact PCR had on stutter at these loci?

Response. The reviewer raises a good point.

- In the current literature, stutter error is generally observed for short tandem repeats (with shorter motifs) during PCR amplification. The error is more prominent as the repeat unit (motif) length decreases, and most pronounced when performing PCR for dinucleotide and mononucleotide repeats (Ellegren, 2004*). Loh *et al* calculated the stutter error in microsatellites (short tandem repeats or STRs) in experiments with targeted sequencing with hybridization and PCR (Loh, 2024*). In Figure 2C of their publication, they showed that stutter is predominant in STRs with motifs of length 2bp, present but less prevalent in STRs with motifs of length 3bp and minimal in STRs with motifs of length 4bp (Loh, 2024*). Therefore, as motif length increases, the stutter error decreases in the hybridization targeted approach with PCR. Here, we considered VNTRs with minimum motif length of 6bp, suggesting that we do not expect stutter errors to be dominant in VNTRs.
- We recognize that our study utilized targeted sequencing data that was not designed with a focus on computing stutter errors. Specifically, the loci did not include male chrX TRs where a single allele is expected and all other lengths can be attributed to stutter error.
- In the discussion, we have added the following text:
  - "In this study, we did not compare differences in stutter errors between targeted and whole genome sequencing. Testing for stutters is typically performed on chromosome X of male samples, where any genotype other than the mode is likely to be a stutter error. Our targeted study samples were used in other studies with Illumina sequencing[38], and no chrX TRs were targeted. It is possible that the accuracy of genotyping targeted VNTRs could be impacted by stutter errors arising during PCR. Published reports on Short Tandem Repeats (STRs) with motif length <= 4, however, suggest that stutter errors have a strong negative correlation with motif length, and they are uncommon in motifs of length 4 [37]. In contrast, our study exclusively looked at VNTRs where the motif length was at least 6 bp. For longer motifs, stutter errors are unlikely to change motif counts, and AdVNTR is robust to small changes in motif sequences, because it uses

underlying Hidden Markov Models (HMMs) allowing insertions and deletions within each motif."

- Instead, we provided aligned read lengths and repeat counts for a few example VNTRs and the sample with id 7 in the targeted cohort in figures RS1 to RS4 below. To maintain conciseness, we are providing figures RS1 to RS4 here but have not added them to the revised manuscript. As seen in figures RS1 to RS4, the repeat counts inferred from the read lengths by AdVNTR has increased consistency when compared to the read lengths (cut to the VNTR boundary) for the same sample and VNTR. This is due to the HMMs in AdVNTR designed to accurately match the motifs even with a slight difference in length (i.e. short indel in the motif).
- Another observation is that as the motif length increases (motif lengths decrease from RS1 to RS4), the erroneous reads where the read length is uncommon (i.e. < 10%) decrease. This is in line with the findings in the literature referenced above.
- *References:

    - Ellegren H. Microsatellites: simple sequences with complex evolution. Nature reviews genetics. 2004 Jun 1;5(6):435-45.
    - Loh CA, Shields DA, Schwing A, Evrony GD. High-fidelity, large-scale targeted profiling of microsatellites. Genome Research. 2024 Jul 1;34(7):1008-26.

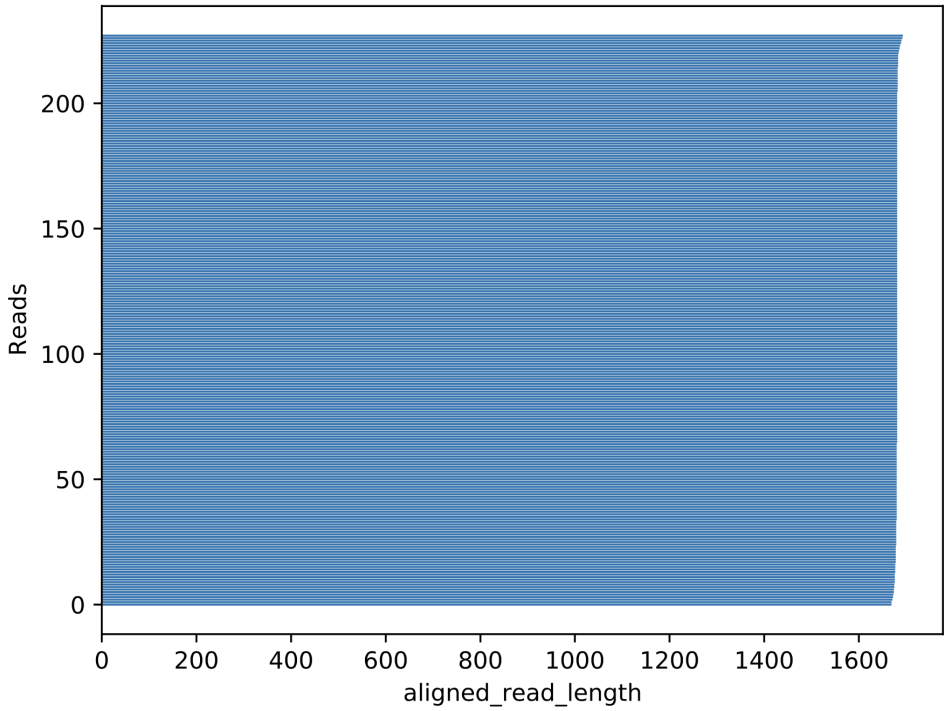

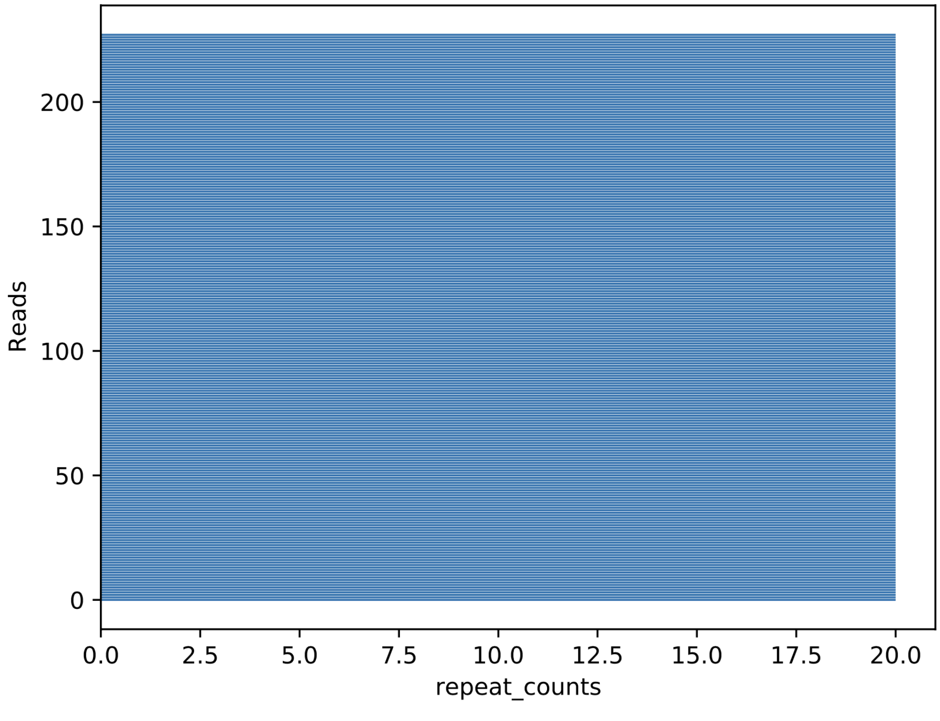

- Supplementary figure RS1. Read length and repeat counts plot for sample id 7 in the targeted sequencing cohort. Showing all the recruited spanning reads for VNTR with motif length 84 bp and GRCh38 reference length of 552 bp. **Top:** The reads are cut to the VNTR boundary. **Bottom:** Repeat counts are inferred using AdVNTR.

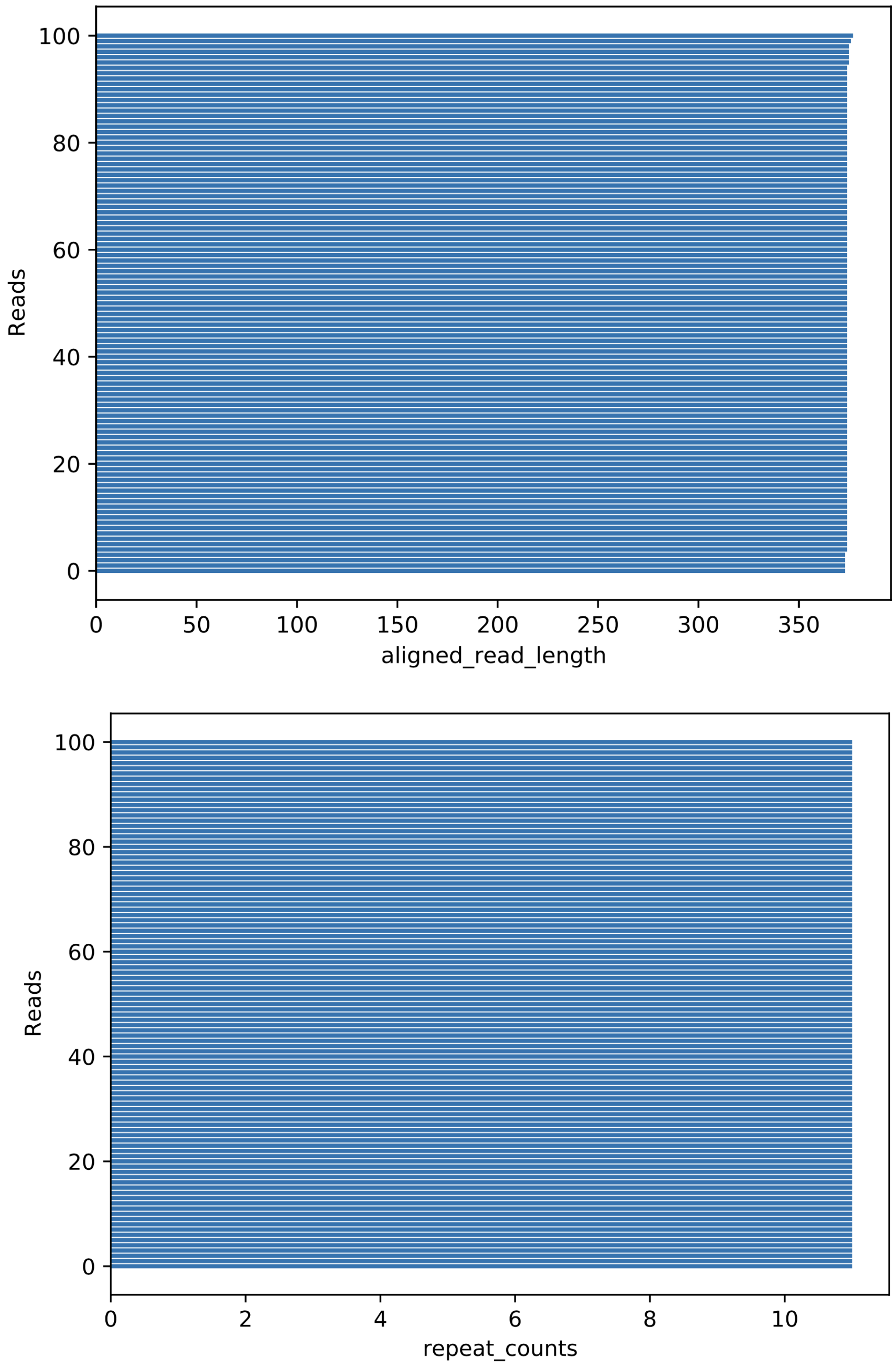

- Supplementary figure RS2. Read length and repeat counts plot for sample id 7 in the targeted sequencing cohort. Showing all the recruited spanning reads for VNTR with motif length 34 bp and GRCh38 reference length of 374 bp. **Top:** The reads are cut to the VNTR boundary. **Bottom:** Repeat counts are inferred using AdVNTR.

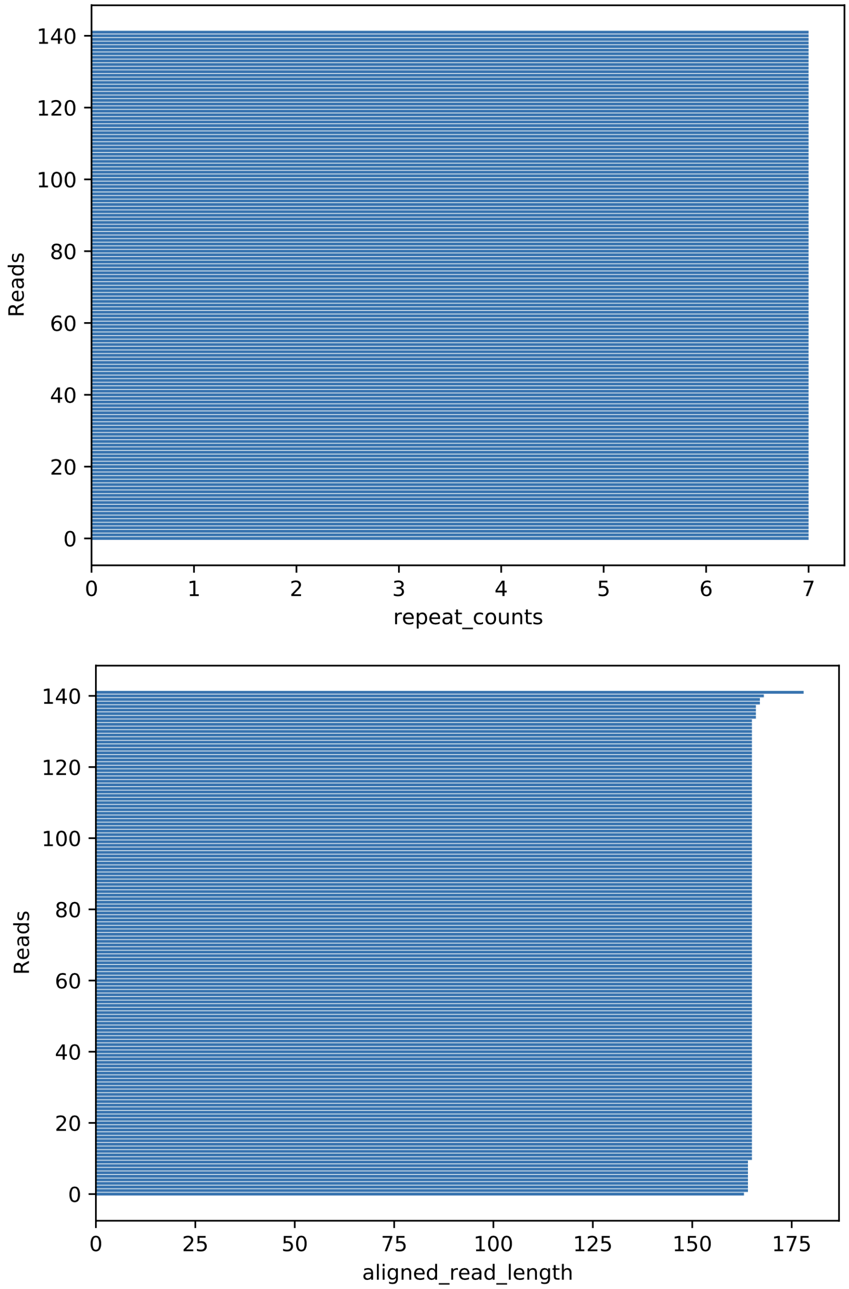

- Supplementary figure RS3. Read length and repeat counts plot for sample id 7 in the targeted sequencing cohort. Showing all the recruited spanning reads for VNTR with motif length 24 bp and GRCh38 reference length of 165 bp. **Top:** The reads are cut to the VNTR boundary. **Bottom:** Repeat counts are inferred using AdVNTR.

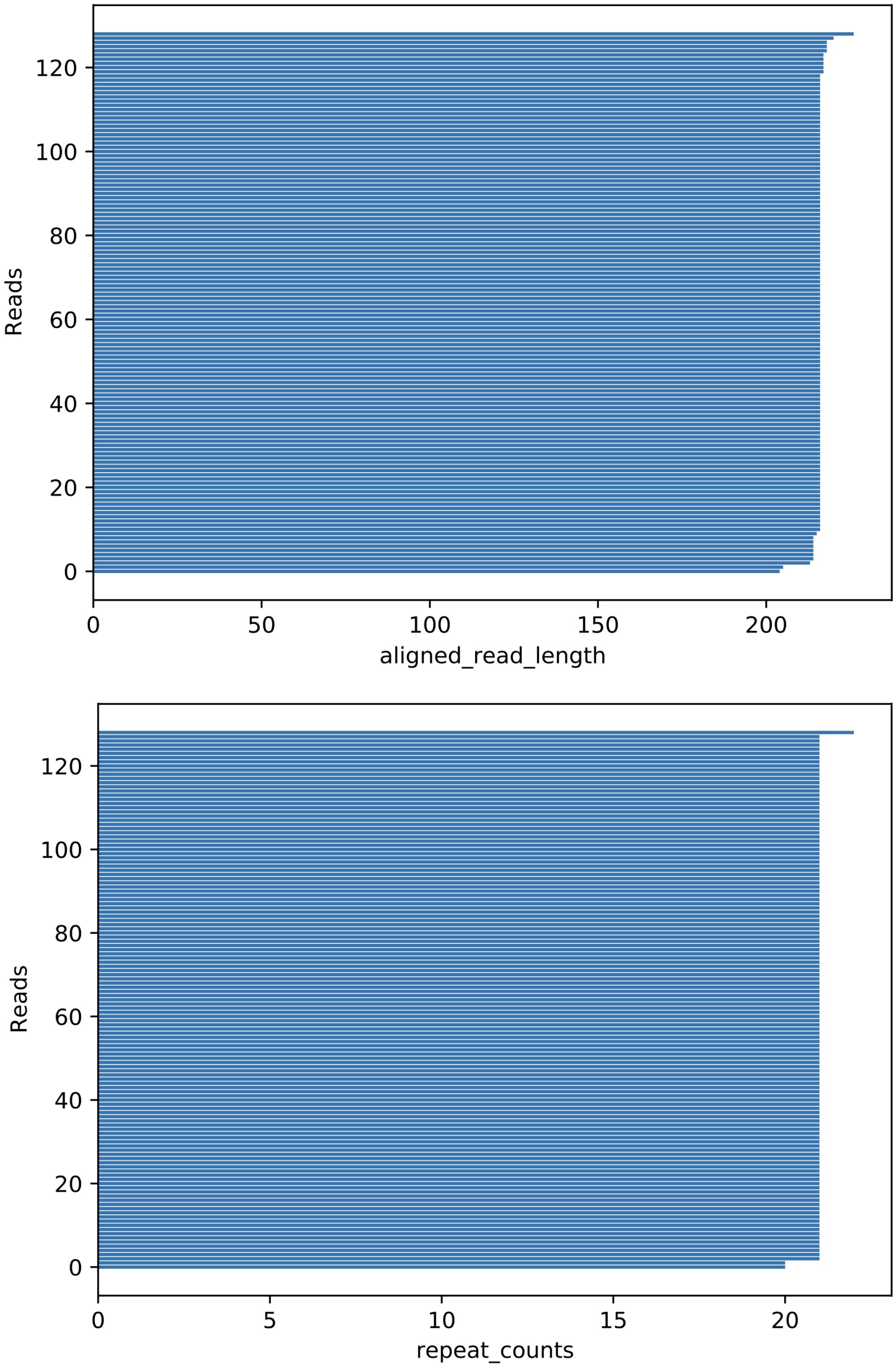

- Supplementary figure RS4. Read length and repeat counts plot for sample id 7 in the targeted sequencing cohort. Showing all the recruited spanning reads for VNTR with motif length 10 bp and GRCh38 reference length of 216 bp. **Top:** The reads are cut to the VNTR boundary. **Bottom:** Repeat counts are inferred using AdVNTR.

Data availability statement needed.

Response. We have uploaded the targeted sequencing Fastq files on the Sequence Read Archive (SRA) with Bioproject number PRJNA1209963 and scheduled to be released on February 20, 2025 with the reviewer link:
https://dataview.ncbi.nlm.nih.gov/object/PRJNA1209963?reviewer=68lgek4bkk5ov6vfkk5bjglk5k

Data availability statement is added including information on the whole genome and targeted sequencing data

- Whole genome sequencing data is publicly available through the Human Pangenome project [34] through the portal https://humanpangenome.org/data/. Targeted sequencing data generated in this study is available on Sequence Read Archive (SRA) with Bioproject number PRJNA1209963.

Minor comments:

“Variable Number Tandem repeats (VNTRs) refer to repeating motifs of size greater than five bp”
The definition of VNTR here is somewhat non-standard, although I acknowledge that there is uncertainty about this in the literature. Some of the “P-VNTRs” of <= 6bp motifs would be more typically referred to as short tandem repeats (STRs). Two have motifs <5 bp, so defy the introduction here as well.
Examples:
FXN - GAA
CNBP - CAGG
NPO56 - GGCCTG
C9orf72 - GGCCCC
It would probably be more accurate to say the majority of targeted loci were VNTRs with some STRs added, and explain why those were chosen to include as opposed to other longer motif pathogenic STR loci such as BEAN1 (TGGAA), RFC1 (AAGGG), STARD7 (AAATG), TNRC6A (TTTCA), or SAMD12 (TGAAA).

Response. The reviewer has an important observation on the repeat unit (motif) of several targeted P-VNTRs. For VNTR within the FXN gene, we would like to point out that there are two overlapping TRs according to the Tandem Repeat Finder (TRF), one with motif (CTT) and one with motif (TCCTTC). Please see Figure R3 below. We selected the latter, with motif length 6bp. Similarly, for the P-VNTRs within the CNBP gene, multiple overlapping TRs with motifs (CAGG, CAGACAGG, CAGACAGGCAGG) indicated in TRF. We selected the VNTR with motif CAGACAGG in this case (see Figure R4).

- For NOP56 and C9orf72 VNTRs, we would like to highlight that the motif length threshold that differentiates STRs and VNTRs can vary in different studies, especially for the motif length 6bp which is at the border of the threshold. Therefore, it is likely that TRs such as the NOP56 and C9orf72 TRs are classified as STR in one study and as VNTR

in another study. That could explain why the reviewer claims that “Some of the ‘P-VNTRs’ of <= 6bp motifs would be more typically referred to as short tandem repeats (STRs)”. The two TRs NOP56 and C9orf72 pointed out by the reviewer have motif length of 6bp each, both meeting the criteria we defined for the VNTRs in our study.

- We decided to focus on VNTRs in this study with the motif length criterion of at least 6bp this excludes the tandem repeats the reviewer suggested (BEAN1, RFC1, etc.) all having motif counts 5bp.
-

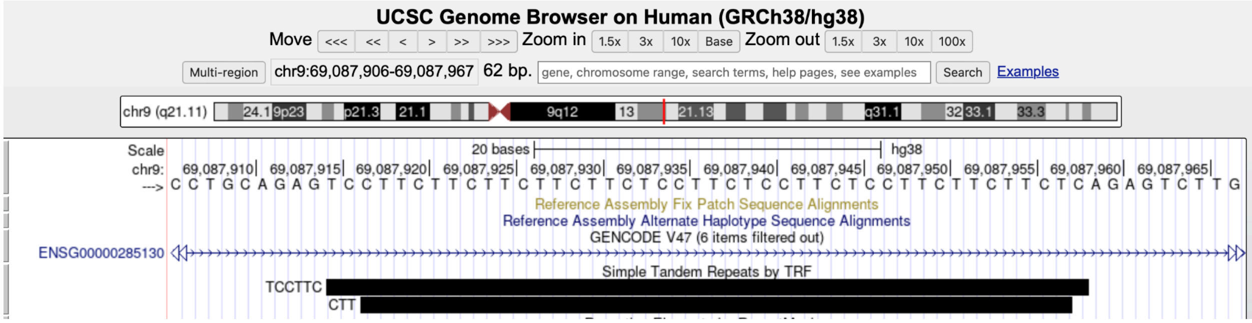

Figure. R3 Genome browser screenshot for P-VNTR in FXN gene

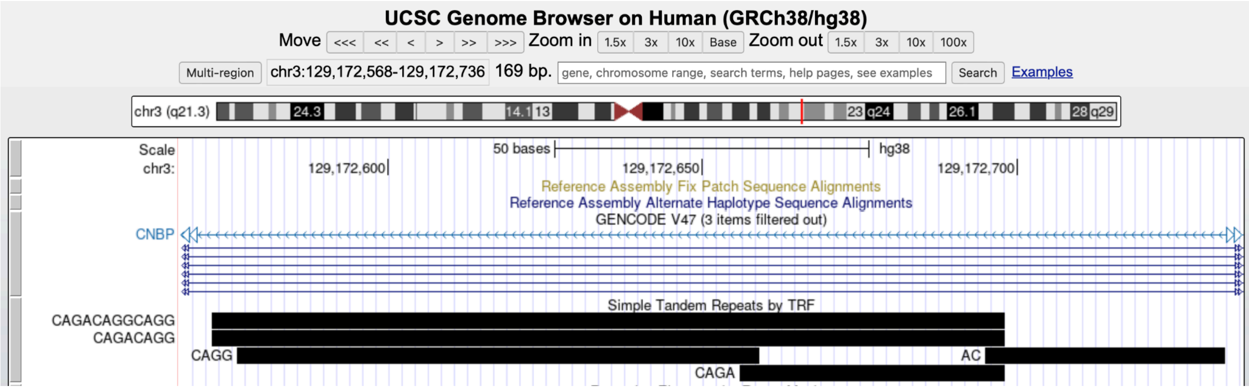

Figure. R4 Genome browser screenshot for P-VNTR in CNBP gene

Mention the targeting method in the abstract for additional clarity. One can infer from the term “custom probes”, but better to be explicit.

Response. We have updated the manuscript as follows:

- We **performed targeted sequencing with hybridization by designing** custom probes for 9,999 VNTRs and sequenced 8 samples using HiFi and Illumina sequencing, followed by adVNTR genotyping.

You could contrast probe design strategy with short read studies, for example: https://genome.cshlp.org/content/34/7/1008

The study by (Loh, 2024*) is a comprehensive and valuable analysis for targeted sequencing using hybridization and Illumina reads on a desired set of microsatellites. However, there are important differences in the problem definition and the purpose of this study and our manuscript. Loh *et al* aimed to provide a framework that minimizes stutter artifacts in microsatellites, and suggest a design strategy for selecting such STRs, based on passing filters. The full list of filters in the Loh *et al* paper as appears in the manuscript is as follows:

- "The full set of requirements for loci in the pilot panel was as follows: (1) total microsatellite length ≤100 bp, (2) period size between 2 and 4 bp, (3) percent match ≥90%, (4) percent indel ≤10%, (5) uninterrupted copy number of four or more, (6) distance to the nearest microsatellite in both directions ≥100 bp, (7) GC content in both the microsatellite and its flanks ≤70%, (8) 50 k-mer mappability score in both 90 bp flanks ≥0.95 (note that the 50 k-mer mappability was used to ensure uniqueness of the 60 bp flanking regions targeted by strategy 3), (9) single-cell PTA normalized coverage (see above) between 0.4 and two in both flanks to target more uniformly amplified regions that may be useful in future single-cell applications, (10) neither 90 bp flank overlaps either the 1000 Genomes Project pilot accessibility mask (regions that are challenging to resolve by short-read sequencing) (The 1000 Genomes Project Consortium 2015) or the ENCODE Data Analysis Center (DAC) blacklist regions (regions that are prone to artifacts and erroneous signal in short-read sequencing) (Amemiya et al. 2019), and (11) neither 90 bp flank or the microsatellite overlap annotated segmental duplications (Supplemental Fig. S3; Bailey et al. 2002)."

In contrast, we start with a set of gene-proximal VNTRs (G-VNTRs) and phenotype-associated VNTRs (P-VNTRs) that are either (or likely to be) clinically relevant and ask if they can be targeted. Indeed, many of these VNTRs did not meet the filtering criteria outlined in the Loh *et al.* paper. Moreover, the motif lengths of VNTRs exceed what is presented in the Loh study. We designed probes using standard Agilent SureDesign and analyzed the results on our set of target VNTRs.

In the revised manuscript, we have added the following text:

- "While our approach focused on VNTRs with clinical relevance, a contrasting approach was followed in a related publication [37], where they focused on a larger set of microsatellites with short motif lengths and deployed a number of filters to enrich for microsatellites that are amenable to targeted sequencing."
- *References:
  - Loh CA, Shields DA, Schwing A, Evrony GD. High-fidelity, large-scale targeted profiling of microsatellites. Genome Research. 2024 Jul 1;34(7):1008-26.

Fig S1, S2 Looks like overplotting could be an issue, especially at zero. Suggest adding jitter or experimenting with other plot styles that can better show density.

Response. This is a very good point. We considered the advantages and disadvantages of adding jitter to the plot and alternative approaches to resolve overplotting. While adding jitter is a great way to fix overplotting in a generic plot, in our specific plots S1 and S2, there is a high density of points in particular horizontal and vertical lines (e.g. SR=0) and we found that adding jitter might not fully reflect the density of the points in those highly dense parts of the plot. Moreover, without the jitter, the spanning reads are visible as discrete values (straight horizontal or vertical lines) in the plot, which is perhaps more accurate.

To resolve these concerns, we updated figures S1 and S2 by adding a separate subplot where the opacity of the points is at 0.1, making the density more visible. The points with 0 spanning reads are dense and not fully visible even with lower opacity. Therefore, we added a separate subplot to show the distribution of points for all the points at spanning reads = 0.

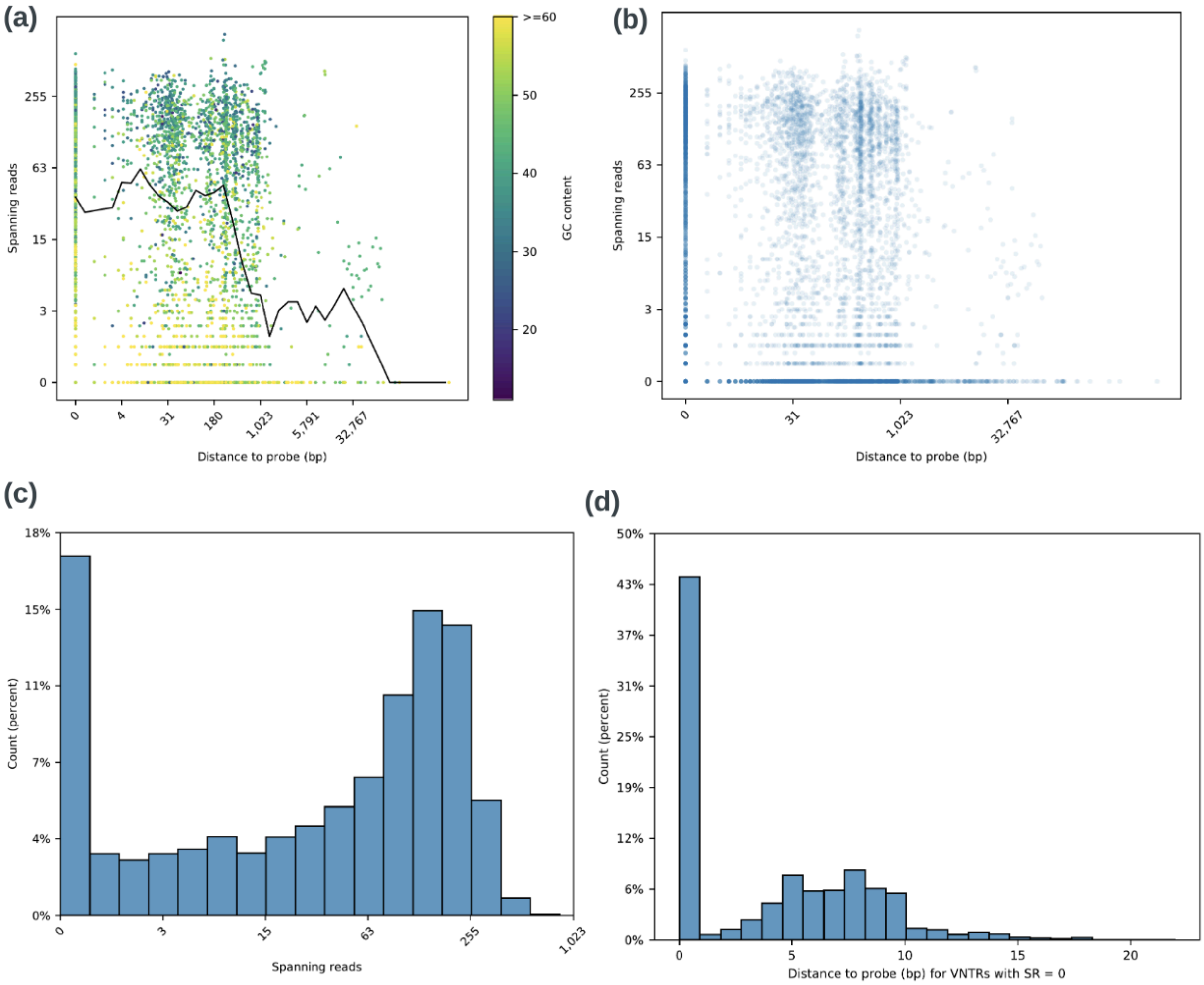

Revised figure S1

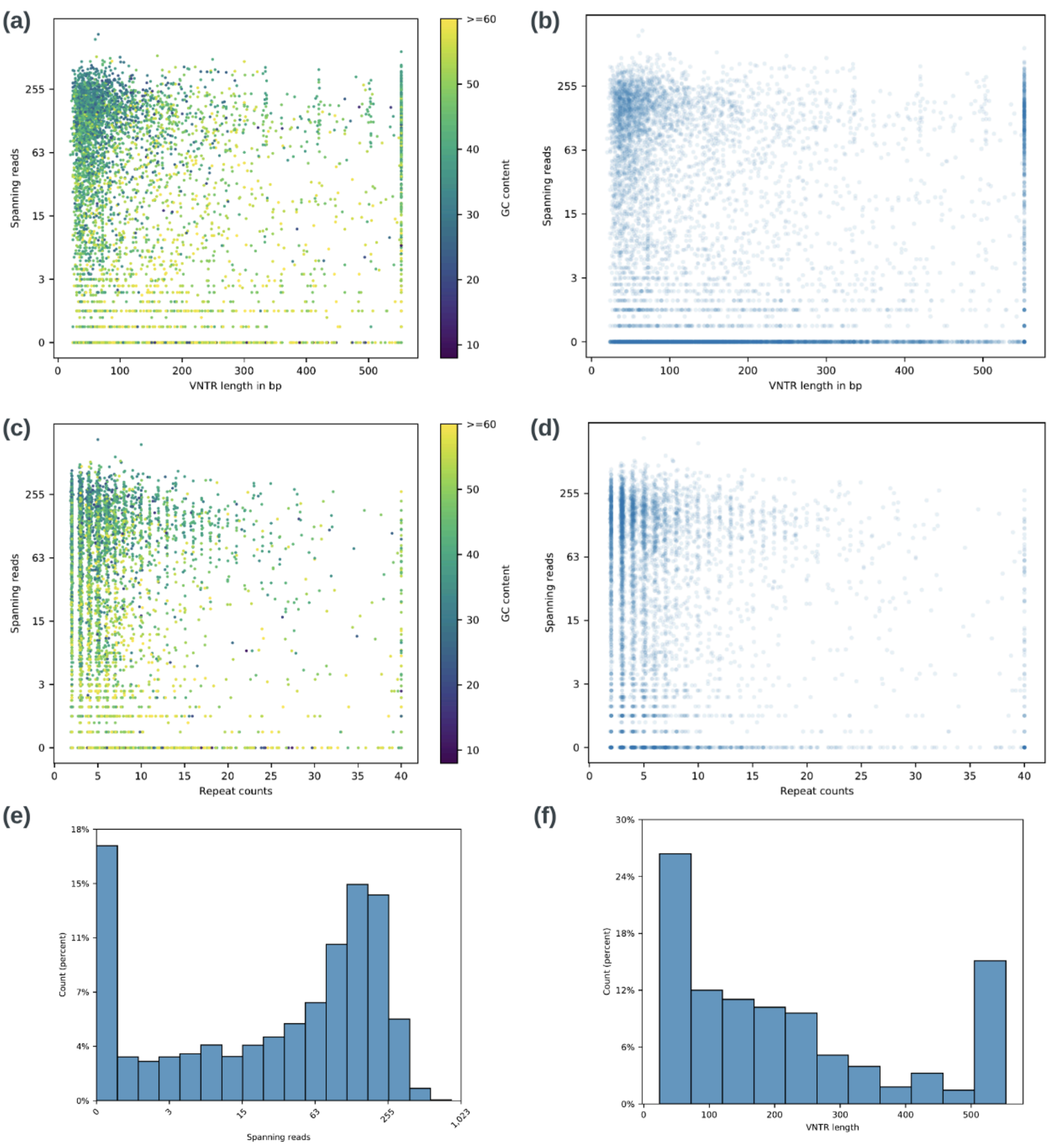

Revised figure S2

In the results section on the CEL VNTR, please briefly mention why you focus on this locus. Would it be possible to disentangle the pseudogene reads, and if so how? What are the implications for prior/future work on this locus?

Response. We thank the reviewer for this comment. We decided to remove the paragraphs on the *CEL* VNTR as it was not in line with the focus of the paper. We wanted to focus on the overall analysis of P-VNTRs and G-VNTRs rather than focusing on a specific VNTR.

"2.2 Targeted Sequencing consistently express low-GC VNTRs"
Seems like there's a word or two missing here? I think what you meant to is that Targeted Sequencing consistently performs better at low-GC VNTRs?

Response. Changes applied:

- Targeted Sequencing consistently performs better at low-GC VNTRs

Section 2.2. Briefly summarize the steps in the target enrichment at the start of the results when it is first introduced. This will give readers to context to understand the relevance of PCR and probe design when it is mentioned later.

Response. That is a great point. We added the following at the beginning of the Section 2.2 in the manuscript:

- Briefly, in targeted sequencing with hybridization, probes are designed for a set of target regions and are hybridized with the target DNA fragments. Magnetic beads isolate the hybridized fragments, separating them from non-target sequences. The enriched DNA is amplified via PCR and then sequenced. More details are available in the Methods section.

Genotyping Validation: I would expect long-read methods to be more accurate than short read here. If you agree, I think you should set this expectation (or explain why it might not be the case). For example, inconsistency could be allelic dropout, or an error in the short-read analysis.

Response. The reviewer is correct in general. Long-read genotyping is more accurate than short-reads mainly because they can more easily span the tandem repeat region with a larger context that ensures correct mapping of the boundaries of the variable tandem repeats. A second factor for correct genotyping is the coverage of the number of reads that span the tandem repeat and ensures that the two alleles are sampled with sufficient redundancy to prevent allelic dropout.

However, because the purpose of our study was to compare targeted versus whole-genome, we first focused on the number of spanning reads while looking only at long-reads. When we wanted to check for genotyping consistency, we compared long and short reads as a validation step and limited the analysis to TRs where both long and short reads could adequately sample the tandem repeat. Therefore, we observed 98% consistency in genotypes. The main reason for inconsistent genotypes is the lack of spanning reads. The most common case in inconsistent genotypes was the case where we have a heterozygous call in one set of reads and a

homozygous call in the other set of reads (what we call “partially consistent” in the manuscript). Partially consistent genotypes add up to 2% of VNTRs. In the fully inconsistent cases, we often observed <15 spanning reads for that VNTR in at least one set of reads.

As discussed in the response to the third comment of the reviewer #2, allelic dropout is unlikely in probe-based enrichment as long as there is sufficient coverage.

We added the following to the “Genotyping Validation” section on the manuscript:

- Other than the number of spanning reads, long reads were expected to provide more context and fully span the longer alleles that do not fit within the short read boundaries.

“Notably, 19% of P-VNTRs involved small changes in repeat motif counts” Typo? Should be something like “pathogenic alleles at P-VNTRs differed by a small change”?

Response. We revised the sentence to:

- Pathogenic alleles in 19% of P-VNTRs involved small changes in repeat motif counts

“Notably, only 4 VNTRs of the 48 P-VNTRs” and “Notably, 23 (48%) came from high GC content regions”. Are both equally notable? Suggest rephrasing to emphasize the key point.

Response. We revised the sentence to:

- Only 4 VNTRs of the 48 P-VNTRs were spanned by ≥ 15 targeted spanning reads. Notably, …

Why did the samples chosen differ between the targeted and WGS approaches? This should be addressed as a limitation, as if some samples overlapped it would be easier to compare directly between sequencing approaches.

Response. We agree that having both targeted and WGS HiFi reads from the same samples would have made the comparison more direct and simpler. However, it was not possible due to sample availability. We have added a paragraph in the discussion section to bring up this limitation (quoted below).

We mainly focused on reporting the number of spanning reads. We observed that the number of spanning reads for a given VNTR across different samples are consistent (see Section 2.2 of the manuscript). Therefore, the number of spanning reads was a feature of a given VNTR rather than a specific sample. In other words, we focused on a metric (spanning reads) where samples were all consistent and therefore the specific sample did not affect the metric.

Comparing genotyping across Illumina and Hifi reads provided additional validation, but we agree that it does not fully substitute for a comparison of targeted and whole genome sequencing. We have added the following on the discussion section of the manuscript:

- "An important limitation of our study is that we did not have matched samples with both whole genome and targeted HiFi reads to facilitate a direct genotyping comparison. This is due to the limited sample availability and the cost of HiFi whole genome sequencing. Here, we focused first on number of spanning reads to allow for a fair comparison, and second, we used multiple datasets, including Illumina and HiFi reads from the same sample to provide genotyping consistency results."

We also added the following in the Section 2.4 of the manuscript

- "Due to limited sample availability and the cost of HiFi whole genome sequencing, we did not have samples with both HiFi WGS and targeted sequencing. As a result, a direct comparison was not possible. Furthermore, the true genotypes for our targeted sequencing cohort were not known. Instead, we genotyped G-VNTRs on multiple datasets where validation was possible, to measure the accuracy of genotyping with spanning reads."

Reviewer #3 - 0/39 comments left

Major comments

The Introduction contains no discussion of previous efforts to detect/genotype VNTRs using whole genome sequencing or targeted sequencing. By "detect" here, I mean determining that the tandem repeats are actually copy number variant. These have been done by several groups. Please include a summary of this work.

Response. We addressed this valid criticism by citing relevant work including (Eslami Rasekh, 2021*), (Ren, 2023*), (Sulovari, 2019*), and (Lu, 2021*) in the introduction section. It is possible, however, that we left out other relevant work and we would appreciate getting pointers to those. The revised text reads as follows:

- TRs have been categorized based on motif length into two classes: 'short' tandem repeats (STRs) when the repeat unit is at most 5-6 bp, and as Variable Number Tandem Repeats (VNTRs) for repeats with longer motifs. We note that different definitions of VNTR have been used in the literature. Eslami Rasekh et al define VNTRs as TRs that are copy number variant in a population[1]. They use the term "minisatellites" to define TR loci with motif length of 7 or greater. In contrast, several others[2–4] define VNTRs as TR's with motif length greater than 6 without requiring evidence of copy number variability. In this paper, we consider VNTR's as all TR loci with a motif length of 6 or more in the reference genome. STRs/VNTRs are among the most polymorphic regions

of the human genome and the polymorphisms manifest as changes in the number of repeat units and less frequently as changes in the repeat unit sequence[5].

- With the availability of whole-genome sequence data, several studies have analyzed the variability of VNTR loci in human populations[1,4,7]. To demonstrate the variability of VNTRs across samples, Lu et al introduced a repeat pangenome graph built on a set of short and long reads sequenced from 19 samples[4]. They found that the graph constructed using 19 individuals is 27% larger than the same graph constructed based on the GRCh38 reference genome[4].

We observed P-VNTRs such as FRA16B, VWA1 (EIF3H, FXN, TAF1) which were classified as low repeat count variation (high repeat count variation) in the pathogenic allele compared to the protective alleles were found to be non-polymorphic in our whole genome dataset. This means that risk alleles could be present in the population, but missing in the healthy individuals in our dataset. We would like to include such VNTRs in our catalog even if they are non-polymorphic in our cohort of healthy individuals.

- * References:

    - Eslami Rasekh M, Hernández Y, Drinan SD, Fuxman Bass JI, Benson G. Genome-wide characterization of human minisatellite VNTRs: population-specific alleles and gene expression differences. Nucleic acids research. 2021 May 7;49(8):4308-24.
    - Ren J, Gu B, Chaisson MJ. vamos: variable-number tandem repeats annotation using efficient motif sets. Genome Biology. 2023 Jul 27;24(1):175.
    - Sulovari A, Li R, Audano PA, Porubsky D, Vollger MR, Logsdon GA, Human Genome Structural Variation Consortium, Warren WC, Pollen AA, Chaisson MJ, Eichler EE. Human-specific tandem repeat expansion and differential gene expression during primate evolution. Proceedings of the National Academy of Sciences. 2019 Nov 12;116(46):23243-53.
    - Lu TY, Human Genome Structural Variation Consortium Munson Katherine M. 2 Lewis Alexandra P. 2 Zhu Qihui 3 Tallon Luke J. 4 Devine Scott E. 4 Lee Charles 3 5 6 Eichler Evan E. 2 7, Chaisson MJ. Profiling variable-number tandem repeat variation across populations using repeat-pangenome graphs. Nature Communications. 2021 Jul 12;12(1):4250.

There is no discussion of testing the tandem repeat reference set to determine if any of the repeats have closely related sequences elsewhere in the genome. Since this is a common occurrence (one example of such an occurrence, the CEL gene VNTR, is mentioned by the authors) and can confound genotyping, this should be carried out and described. If the targeting protocol can be guaranteed to produce unique primers that will not bind to other loci, including segmental duplications, this should also be reported.

Response. We thank the reviewer for bringing up this point. In the original selection of VNTRs, we excluded G-VNTRs that were located in known regions of segmental duplications. In the revised manuscript, the following is added to Methods:

- We started with tandem repeats identified by Tandem Repeat Finder [41] on the GRCh38 human reference genome and limited those to TRs with motif length of at least 6 and excluded VNTRs within LINE and SINE elements. We also excluded VNTRs within the segmental duplicated regions to ensure that the recruited reads were, in fact, from the target region.
- For P-VNTRs, we added a column in the supplemental table S2 to indicate whether or not the P-VNTRs are located in a segmental duplication region.

Please describe how many of the genotyped G-VNTRs were found to be heterozygous in at least one genome, in more than one genome. That is, are they actually VNTRs (i.e., copy number variant)?

Response. The percentage of non-polymorphic P-VNTRs and G-VNTRs in the WGS cohort is 10% and 76% respectively. The reviewer has a valid point that variability is a key part in selecting VNTRs of interest. However, we could not confidently discard all VNTRs that were non-polymorphic in our small cohort because they could vary in other individuals. For example, P-VNTRs FRA16B, VWA1, EIF3H, FXN, TAF1 for which repeat count variation was observed in risk and protective alleles in literature, were found to be non-polymorphic in the WGS dataset in our study. This suggests that even though these VNTRs are non-polymorphic in the WGS cohort of healthy individuals, risk alleles could be present in the population, specifically among the individuals affected by the phenotype. As a second point, the conclusions of our study are based on counts of spanning reads and should hold for most VNTR subsets. We have added the following text in the Results section:

- Polymorphism in targeted VNTRs. While the interesting VNTRs were more likely to be polymorphic in general, 5 of our P-VNTRs (FRA16B, VWA1, EIF3H, FXN, TAF1) were non-polymorphic in the WGS cohort, but are reported to be copy number variant in the literature (see Table S2). Similarly, we observed that 76% of G-VNTRs were non-polymorphic in the WGS cohort, but a larger fraction may be polymorphic in a larger, more diverse population or among non-healthy individuals. Our conclusions, based on the counts of spanning reads, are likely to hold in most cases.

What was the distribution of allele motif copy numbers for all the G-VNTRs. This is not addressed by Figure S2, which groups TRs by motif length.

Response. We have added the distribution of the repeat counts to the figure S2. The distribution of repeat counts is very similar to the VNTR length distribution. We would like to mention that VNTR length provides a more spread out scatter plot compared to the repeat count plot.

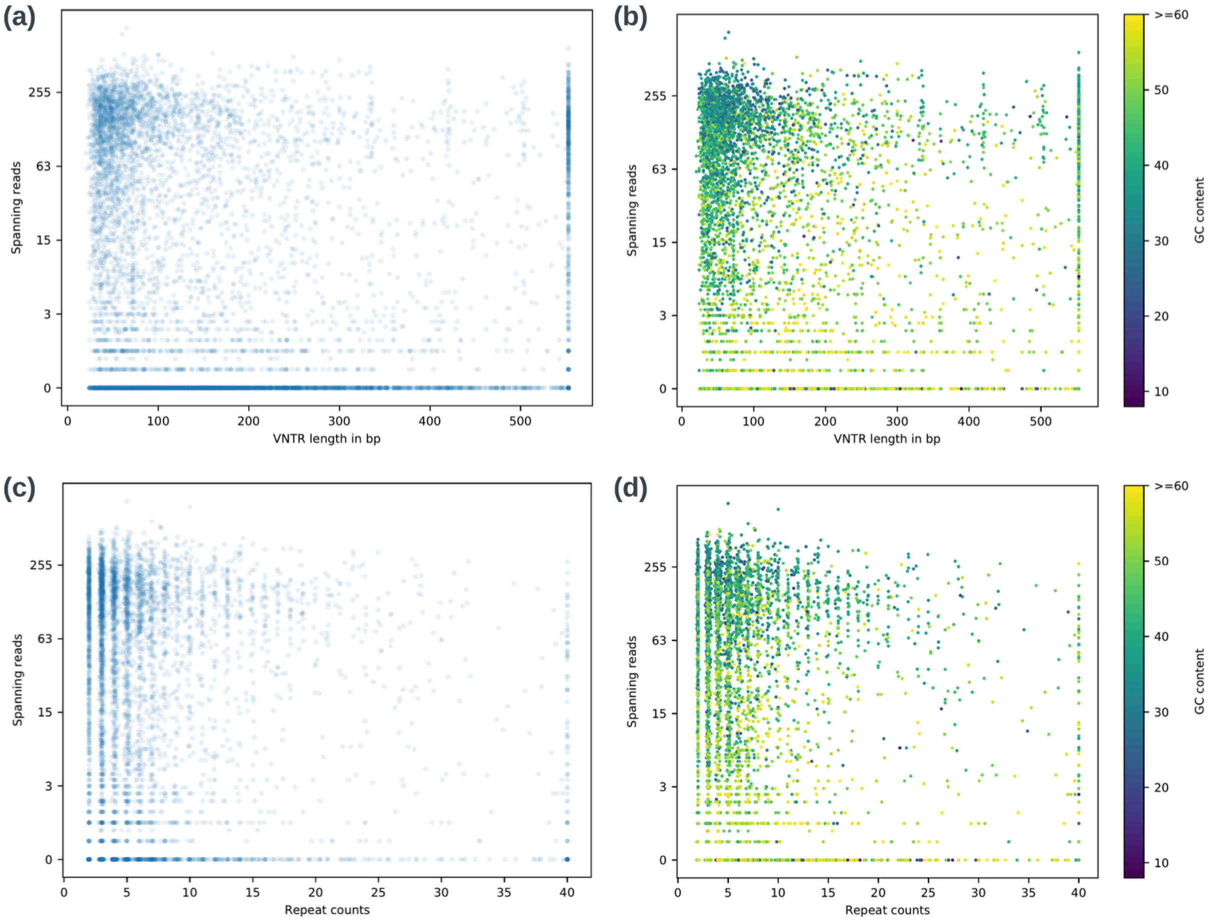

- Updated Figure S2

****

Minor comments/editing suggestions
Below I list editing comments and questions. Each part is preceded by the relevant page number/line number. My comments are preceded by >>.

--- Abstract ---
Variable Number Tandem repeats (VNTRs) refer to repeating motifs of size greater than five bp.
>>This is an inaccurate statement. VNTRs refer to tandem repeats that are variable in copy number. Not all repeats with motifs size greater than five are variable in copy number. The class of tandem repeats you describe are called minisatellites. Please rephrase and change the wording throughout the manuscript.

Response. We understand the reviewer's comments about the variability in the VNTRs and we also addressed this issue when it was first mentioned as the first major point for this reviewer. However, replacing the term "VNTR" with "minisatellites" could also be misleading for readers.

The term VNTRs, somewhat unfortunately named, appears to be commonly used to describe the tandem repeats with higher motif lengths, located in Euchromatin regions. See for example (Ren 2023*). Minisatellites are often used to refer to repeats predominantly located in centromeric and telomeric regions, but indeed have been used in this context (Eslami Rasekh 2021*). Some authors have acknowledged this and use both (Sulovari 2019*).

In addition, as we mentioned in the response to the first major point of this reviewer, the confusion arises from the fact that variability of VNTRs is not easy to define. Some VNTRs that are not variable in one cohort could be variable in a more diverse cohort with a variety of backgrounds and phenotype/disease status.

We added the following to the introduction:

- TRs have been categorized based on motif length into two classes: ‘short’ tandem repeats (STRs) when the repeat unit is at most 5-6 bp, and as Variable Number Tandem Repeats (VNTRs) for repeats with longer motifs. We note that different definitions of VNTR have been used in the literature. Eslami Rasekh et al define VNTRs as TRs that are copy number variant in a population[1]. They use the term “minisatellites” to define TR loci with motif length of 7 or greater. In contrast, several others[2–4] define VNTRs as TR’s with motif length greater than 6 without requiring evidence of copy number variability. In this paper, we consider VNTR’s as all TR loci with a motif length of 6 or more in the reference genome. STRs/VNTRs are among the most polymorphic regions of the human genome and the polymorphisms manifest as changes in the number of repeat units and less frequently as changes in the repeat unit sequence[5].
- With the availability of whole-genome sequence data, several studies have analyzed the variability of VNTR loci in human populations[1,4,7]. To demonstrate the variability of VNTRs across samples, Lu et al introduced a repeat pangenome graph built on a set of short and long reads sequenced from 19 samples[4]. They found that the graph constructed using 19 individuals is 27% larger than the same graph constructed based on the GRCh38 reference genome[4].

* References

- Eslami Rasekh M, Hernández Y, Drinan SD, Fuxman Bass JI, Benson G. Genome-wide characterization of human minisatellite VNTRs: population-specific alleles and gene expression differences. Nucleic acids research. 2021 May 7;49(8):4308-24.
- Ren J, Gu B, Chaisson MJ. vamos: variable-number tandem repeats annotation using efficient motif sets. Genome Biology. 2023 Jul 27;24(1):175.
- Sulovari A, Li R, Audano PA, Porubsky D, Vollger MR, Logsdon GA, Human Genome Structural Variation Consortium, Warren WC, Pollen AA, Chaisson MJ, Eichler EE. Human-specific tandem repeat expansion and differential gene expression during primate evolution. Proceedings of the National Academy of Sciences. 2019 Nov 12;116(46):23243-53

with at least 15 spanning reads, albeit with lower coverage.
>>Why is 15 the criterion?

Response. This is a very good point. We added the following to the manuscript (Methods).

- To compute a threshold for spanning reads, we estimated the probability of incorrectly calling a heterozygous genotype as homozygous, given *n* spanning reads. Such an incorrect call in AdVNTR occurs when there are 2 or fewer spanning reads from one allele. Considering a case with *n* total reads, *x* reads sampled from one allele, and no allele dropout

$$P_{\text{err}}(n) = P(x \geq n-2 \text{ OR } x \leq 2) = \sum_{i=n-2}^{n} \binom{n}{i} \cdot 0.5^{i} \cdot 0.5^{n-i} + \sum_{i=0}^{2} \binom{n}{i} \cdot 0.5^{i} \cdot 0.5^{n-i} \quad (1)$$

From Eqn. 1, P_err(14) = 0.013, P_err(15) = 0.0074. Thus, n = 15 is the lowest number of reads where the probability of miscalling a heterozygous genotype is lower than 1%, and it was chosen as the threshold for spanning reads.

--- Intro ---
1/3
Functionally, TRs have been classified as ‘short’ tandem repeats (STRs), when the repeat unit is at most 5 bp, and as Variable Number Tandem Repeats (VNTRs) when 4 the repeat unit is at least 6 bp.
>>This is an inaccurate statement. VNTRs refer to tandem repeats that are variable in copy number. Not all TRs with motifs size greater than five are variable in copy number. The class of tandem repeats you describe are called minisatellites. Please rephrase and change the wording throughout the manuscript.

Response. Yes, the reviewer makes a valid point, but there is some confusing terminology in the published literature. Please see our response above to an identical comment.

2/25
The problem is compounded for VNTRs within low copy or segmental duplication regions.
>>clarify "low copy" here

Response. We have removed the “low copy” keyword.

2/49
we systematically explored the tradeoffs between targeted sequencing via hybridization and

whole genome sequencing for VNTR genotyping
>>The manuscript describes one moderately effective targeting protocol and one WGS data set. This does not seem to be systematic.

Response. We have removed the word "systematic".

--- Methods ---
Various places on page 12:
at the recommended settings
using the recommended conditions
etc.
>>these phrases appear in various places in the DNA preparation steps. Please give references to where these recommendations are detailed.

Response. We updated the manuscript in 3 instances where "recommended conditions" or "recommended settings" was mentioned:

- DNA damage repair, end-repair/A-tailing, and adapter ligation steps were performed on the captured samples using the SMRTbell Express Template Prep Kit 2.0 (Pacific Biosciences) **following the manufacturer's specifications.**
- 2 µg of DNA from each sample was sheared to a target size of 6 kb using g-TUBEs (Covaris) **with RPM of 7000 and spin time of 2 minutes**.
- The sheared DNA for each sample underwent end repair and A-tailing with the KAPA Hyper Prep Kit **following the manufacturer's specifications** (Roche).

13/311
We started with 10,787 gene proximal VNTRs (G-VNTRs) which are defined by VNTRs within the gene boundaries and up to 500bp of the transcription start site (TSS) which account for promoter and other regulatory regions.
>>How was the original list of TRs obtained? You describe a selection or filtering process to obtain the G-VNTRs, so there must have been an original list. Where did it come from? Please describe and include a citation.
>>Was there prior evidence that the G-VNTRs were actually copy number variant?

Response. We updated the Methods section to describe how the list of TRs are obtained:

- We started with tandem repeats identified by Tandem Repeat Finder [41] on the GRCh38 human reference genome and limited those to …

We have addressed the concerns about G-VNTRs and P-VNTRs being copy number variant in the first minor comment of the same reviewer #3 in the abstract section. Not all G-VNTRs are copy number variant in the WGS cohort.

>>Please detail how the downstream "gene boundary" is defined
>>Regulatory regions for genes could be further away than 500bp and 25 of your P-VNTRs are not in this list, so presumably further than 500 bp away from some gene. Why was 500 bp chosen? Please justify.

Response. Gene boundaries are extracted from the 2019 RefSeq NCBI gene annotations. This is added to the manuscript as well:

- Gene annotations are extracted from the 2019 RefSeq NCBI gene annotations

The reviewer has a good point about distant gene regulatory elements. While promoters are typically located upstream of the gene, gene regulatory elements could be thousands of nucleotides away from the gene boundary. However, we limited the VNTRs of study to those within 500bp upstream of the TSS. Because of the cost and resources to perform targeted sequencing with custom probes, we had to limit our set of target VNTRs and we chose to do so by limiting the VNTRs to be within gene boundaries or 500bp upstream of TSS.

We agree that our set of G-VNTRs might not encompass all the gene regulating VNTRs. That could be one reason why P-VNTRs and G-VNTRs do not fully overlap. However, we believe the G-VNTRs could be a great resource for researchers who would like to start working with a limited set VNTRs (10,787 VNTRs here) that are within the gene boundaries or otherwise are likely involved in gene regulatory elements e.g. promoters.

The P-VNTRs that are not in G-VNTR could be due to being located further than 500bp away from the gene boundaries (e.g. EIF3H VNTR). But other reasons could cause that including being located in segmental duplication regions, SINE and LINE elements (see Supplementary table S2, column “Is within segdup regions”). The CEL, PRDM9, MUC1, and DUX4 VNTRs are good examples of this case.

We added the following to the discussion section of the manuscript:

- VNTRs that lie in segmental duplications can be challenging to genotype correctly. For this study, we identified and filtered G-VNTRs that were located in segmental duplications (Methods). However, P-VNTRs were selected based on the literature and four (PRDM9, MUC1, CEL, and DUX4) were located in the segmental duplicated regions (see Supplementary Table S2). Targeted sequencing was only successful where there were at least 15 spanning reads for the PRDM9 VNTR, for which we verified that the reads correctly mapped to the locus.

13/315
The database of G-VNTRs is available online at the AdVNTR Github directory.
>>What is the file name for the G-VNTR set in that directory? I tried looking but couldn't determine the correct file.

Response. We provided the full link in the manuscript (Methods, Section 4.2) and specified the exact section to find the database:

- The database of G-VNTRs and P-VNTRs is available online in the AdVNTR Github directory: https://github.com/mehrdadbakhtiari/adVNTR under "Data Requirements and Pre-trained Models (Databases)".

13/317
whole genome PacBio HiFi reads already mapped to the human reads using Winnowmap
>>Is "human reads" a typo?

Response. We have corrected it as follows:

  - whole genome PacBio HiFi reads already mapped to the human **reference genome GRCh38** using Winnowmap

13/318
Furthermore, we utilized the whole genome Illumina sequencing reads from the same cohort alongside the parent short read data for the genotyping validation step (see Sections 2.4 and 4.2).
>>What is the parent short read data?

Response. We have clarified it as follows:

- Furthermore, we utilized the whole genome Illumina sequencing reads from the same cohort alongside the **whole genome Illumina sequencing reads for the parents of the individuals (when available)**

13/322
However, one sample with id 40431 was excluded from analysis due to low coverage across all targeted regions.
>>Does this mean you used only 7 samples, not 8 in the remainder of the manuscript? You consistently refer to 8. Please clarify.

Response. The reviewer brings up a very good point. The referenced sentence is removed from the manuscript in the latest edit as that particular sample proved to have coverage similar to other samples. See Supplementary table S4 for the number of sequenced reads (sample 40431 is mapped to sample id 7 in the updated version).

>>from table S4, sample 40566 had low overall coverage (about 20% of the median across all samples). Did this one have sufficient coverage across the targeted regions? Please define "low coverage."

Response. Sample 40566 (now referred to as sample 5) has lower overall coverage compared to other samples from the targeted sequencing cohort. This is reflected in Figure 2c of the manuscript, column 5, where the number of spanning reads are color-coded per VNTR. As mentioned above, we decided to remove the statement for sample 40431 including the term “low coverage”.

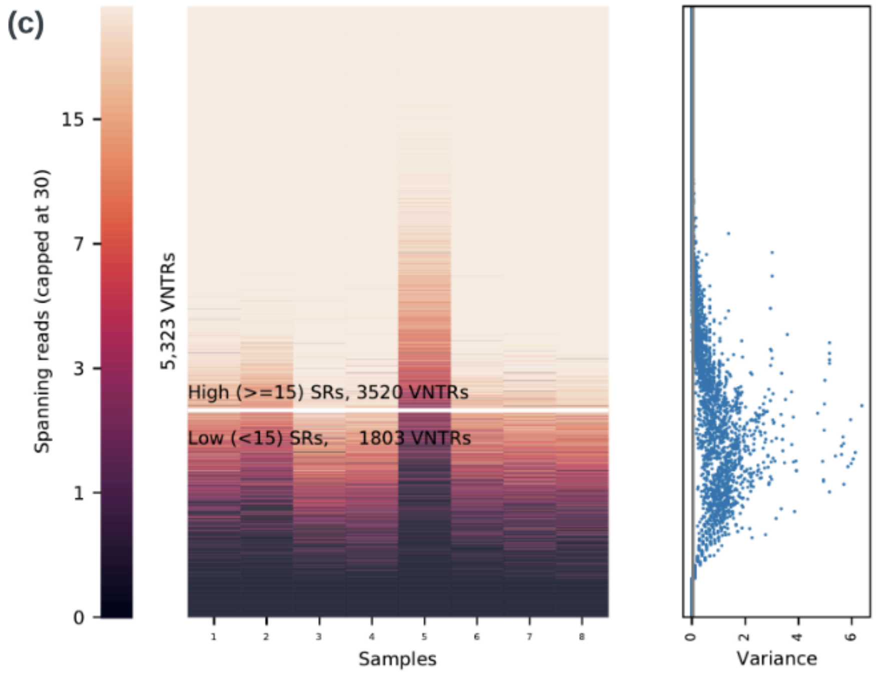

○

- Figure 2c as in the manuscript

13/323
Detailed information about the COH targeted sequencing cohort
>>If the abbreviation means City of Hope, please write it out in full or define the abbreviation earlier.

Response. We have changed it to:

- Detailed information about the **City of Hope** targeted sequencing cohort

13/332
We define reliable supporting reads as reads with at least 95% matching rate in the flanking region and at least 10bp on each of the left and right flanking sides.

>>Just to be clear, is the minimum required flanking region 10bp on each side (the phrasing is not quite clear on this) and does the 95% match apply to this combined minimum 20 bp?

Response. This is a good point. The minimum required flanking region is 10 bp on each side (20bp total). The 95% match criterion is applied for each flanking region separately. We clarified this in the manuscript:

> We define *reliable* supporting reads as reads with at least 10 bp on each of the left and right flanking sides (20bp total) and a matching rate of at least 95% on each of the flanking regions.

>>This is important because the introduction stressed the importance of using flanking sequence to distinguish similar repeats occurring in different regions of the genome, to avoid mischaracterization of genotype. 10 bp is not long enough to distinguish similar repeats in different regions and does not take advantage of long reads extended coverage of flanking regions. Here's the relevant sentence from the Introduction (2/26):

Response. This is a very good point. We agree that 10 bp in each flanking region is not enough to guarantee a read is aligned to only one region. There are a few points we would like to highlight here:

- The issue of recruiting reads by the genotyping tool that are not sequenced from the desired VNTR region seems to be a mapping issue rather than a genotyping issue. Misalignment is less common in long HiFi reads when compared to short Illumina reads (due to the shorter context in Illumina reads). Therefore, our main concern for recruiting reads that were not sequenced from the target region is for short Illumina reads and we are less concerned about misalignment in HiFi reads as they provide a longer context for sequence mapping.
- Our criterion for classifying a read as “reliable” supporting read does not affect sequence mapping. Sequence mapping is previously done by another tool, bwa, prior to calling AdVNTR. We first select mapped reads from the desired VNTR region and later apply the criterion for flanking region to drop the reads that could possibly cause noise in the inferred repeat counts. This is in addition to requiring the read to map to the desired VNTR region. In other words, a read might pass the sequencing mapping criteria (e.g. in BWA) which is a general sequence mapping tool. But we later apply our own flanking filter which is VNTR-specific to further filter the reads that are not likely to help infer the correct repeat counts.
- In all of our genotyping validation experiments we were comparing HiFi reads and Illumina reads for the same sample. Mendelian consistency experiments were also computed on Illumina reads. In other words, one side of the comparison always involved short Illumina reads. For the short Illumina reads, the length of the flanking region we can enforce is limited due to the limited read length. We wanted to keep the genotyping pipeline (including the read recruitment criteria) the same between Illumina and HiFi reads to keep our comparisons consistent.

- We added the following to the manuscript to clarify this:
    - Note that the flanking region criteria are applied to reads that are already mapped to the desired VNTR region. In other words, the flanking region criteria are in addition to sequence mapping requirements and are not replacing them. Moreover, for the genotyping validation and Mendelian consistency experiments in this study, one side of the comparison was short Illumina reads. In order to keep the genotyping pipeline and requirement consistent between short and long reads, we enforced a limited 10 bp flanking region criterion.

For example, the 563 bp VNTR in exon 11 of the CEL gene is not only too long to be spanned by short-reads, but also occurs within a duplicated CELP pseudogene. In these cases, genomic context (i.e. flanking regions) mapped to long spanning reads can provide additional information for reliable mapping
>>As an additional point, was the collection of target TRs screened to determine if they had homologs in other parts of the genome that might cause mischaracterization of genotype? If so, please describe. If not, please justify why not.

Response. We decided to remove the paragraph discussing the CEL VNTR as it was not aligned with the focus of the paper. We wanted to focus on an overview of the P-VNTRs and G-VNTRs rather than focusing on a single specific VNTR.

We excluded the VNTRs that are in segdup regions from our G-VNTRs database. The following is added to Methods:

- We started with tandem repeats identified by Tandem Repeat Finder[41] on the GRCh38 human reference genome and limited those to TRs with motif length of at least 6 and **excluding VNTRs within LINE and SINE elements. We also excluded VNTRs within the segmental duplicated regions to ensure that the recruited reads are, in fact, from the target region.**
- For P-VNTRs which are gathered from the literature, some are in fact within segdup regions and therefore genotyping those VNTRs should be done more carefully. We added a column in the supplemental table S2 to indicate whether or not the P-VNTRs are located in a segmental duplication region.

13/336
For this analysis, we focused on G-VNTRs. To apply this Mendelian consistency analysis
>>Why was validation by Mendelian consistency limited to the G-VNTR group instead of the P-VNTR group as well?

Response. That is a good point by this reviewer. For Mendelian consistency analysis, we focused on G-VNTRs rather than P-VNTRs for multiple reasons:

- G-VNTRs outnumber P-VNTRs and therefore, the addition of P-VNTRs does not affect the overall statistics.
- For Mendelian consistency analysis we focused on VNTRs with length of < 150bp (in GRCh38 reference genome) as the parent data did not include long reads. Therefore, we were looking at P-VNTRs < 150bp which leaves us with 15 P-VNTRs.
- According to the literature, some of the 15 P-VNTRs had large expansions resulting in alleles that are longer than 150bp. We would not have spanning reads for those longer alleles, therefore we could not genotype.

13/343
The STR-like filter for VNTRs
>>When was this filter applied, only for validation or for the entire analysis described in the manuscript? If only for validation why only then? If for the entire analysis, describe when it was applied.

Response. The STR-like filter was applied for the genotype validation step and not the entire analysis. The spanning reads are not affected due to STR-like motifs. However, in genotyping of a VNTR with STR-like motif such as “AAAAG”, a genotyping method might estimate the length of the VNTR by mistake that can cause an inconsistency in the genotyping where in fact the underlying sequences are consistent in terms of repeat counts. Another reason is that due to the STR-like sequence, we hypothesize that stutter error could appear more frequently in these VNTRs and that could cause inconsistency in genotype validation.

We clarified this in the manuscript:

- This filter was only applied to the genotype validation process where the genotype of a VNTR could be incorrectly estimated by the genotyping method and therefore cause an inconsistency where in fact the underlying sequences are consistent in terms of repeat counts.

>>The whole section on STR-like filtering is written in a confusing way and should be rewritten for clarity.

Response. We re-structured this paragraph in the manuscript. We started with an example and provided an overview of the score function before diving into the details:

- This filter was only applied to the genotype validation process. This is because the genotype of an STR-like VNTR could be incorrectly estimated by the genotyping method and therefore cause an inconsistency where in fact the underlying sequences are consistent in terms of repeat counts. To apply the filter, we computed a score between 0 and 1 for the consensus motif. The premise of the score is to distinguish motifs such as AAAAGA and GGCCTG by assigning a higher score to the former compared to the latter because it is easier to distinguish motifs in the VNTR corresponding to the second motif compared to distinguishing the motifs in the VNTR corresponding to the first motif. To

compute this score, we iteratively masked out characters in the motif and observed if the masked motif included a perfect repeat. For example, masking out the G character in AAAAGA by a character that could match to any other character (AAAA-A), we can find an internal repeat of AAA (matches perfectly to A-A). To find an internal repeat, we computed the Hamming distance between the masked motif against a sliding window of itself. In computing the Hamming distance, we matched the masked character with any other characters i.e. AAA matches with A-A in the example above with a Hamming distance of 0.

13/344
Manhattan distance
14/352
finding the maximum Hamming distance
>>Please clarify which metric is being used and if Manhattan, define in this context. If Hamming distance, wouldn't you want the minimum Hamming distance?

Response. Corrected. We use “Hamming distance” now.

14/358
Finally, the score(M) is normalized by motif length, therefore the values are between [0,1] with score of 1 having an internal repeat with least imperfections
>>Wouldn't a score of 0 have least imperfections?

Response. We thank the reviewer for catching a mistake in describing the score function. We fixed it in the manuscript:

- The normalized score for motif score (M) with length n is computed by finding the maximum similarity (n - Hamming distance).
- Instead of computing the maximum Hamming distance between M’ and a rolling window of size n from M’’, we find the maximum *similarity (n - Hamming distance)* of the two strings.

13/336
To apply this Mendelian consistency analysis on a wider range of trios, we used the HPRC dataset trios with whole genome Illumina short reads and with a focus on target G-VNTRs that are < 150bp.
>>How many trios is this?

Response. As indicated in Section 2.4 of the manuscript under “Mendelian Consistency” paragraph, the data consisted of 28 trios. We repeated this information on the Methods section as well.

14/356
we set n to be the ceil(n/10 if n/leq40 and 1 otherwise.
>>Please reformat for correction and clarity

Response. Fixed.

>>The P-VNTR group was not described in the Methods.

Response. That is a very good point. We mentioned how P-VNTRs are collected in the Methods section:

- P-VNTRs were selected from the literature where VNTR-phenotype associations were previously observed. We additionally applied a criterion on the motif lengths of at least 6bp. The Supplementary Table S1 indicates the VNTR gene name, associated phenotypes, and references.

--- Results ---

4/69
We found similar trends of increased complexity in terms of motif lengths and number of motifs. Specifically, 71% of P-VNTRs and 57% of G-VNTRs, respectively, had repeat unit lengths ≥ 20 bp (Figure 1d), suggesting a more complex motif structure due to an increasing number of motifs with single nucleotide variations.
>>Motif length does not imply complexity. I suggest just stating that many motifs were longer than a certain size. Do you report on the number of motifs with SNPs? If not, drop this comment.

Response. The comment on the complexity of motif structure was removed.

4/72
In contrast with short-read sequencing, PacBio HiFi reads have lengths 10-25 kbp and accuracy higher than 99%, and are capable of spanning all the VNTRs in this study. However, G-VNTRs contribute to only 0.06% of the human genome, motivating the need for more efficient, targeted sequencing.
>>This belongs in the Introduction and I believe is already mentioned there.

- Removed.

4/77

We selected 9,999 G-VNTRs out of 10,787 G-VNTRs that were located on autosomal chromosomes.
>>Does this mean that the unselected G-VNTRs were on the X and Y chromosomes? Or were there other filtering criteria?

Response. There were no other criteria than being on autosomal chromosomes. We clarified the sentence in the manuscript:

- Out of 10,787 G-VNTRs, we selected those located on autosomal chromosomes, resulting in 9,999 G-VNTRs.

4/80
with N50=4,207 bp (Supplemental Table S2).
Does N50 refer to the fragment lengths? State clearly. Also, information on average number of reads is in S4, not S2.

Response. Fixed the Supplementary Table S2 to S4. N50 refers to the read lengths. We clarified this in the manuscript:

    - with **the read length of** N50=4,208 bp

4/83
The coverage was bi-modal in log scale with modes at 0 and 2^7 (Figure 2a).
>>I don't see a number as large as 2^7 for spanning reads in Figure 2.

Response. In Figure 2a in the manuscript, spanning reads are plotted on the X-axis of the center plot and the top histogram (shared X-axis). While 2^7 (or 128) is not explicitly marked, it lies between 64 and 256 on the bottom X-axis. The top histogram shows a sharp peak at *spanning reads = 0,* representing VNTRs without any spanning reads, and a smaller, more gradual peak at 2^7, representing VNTRs with spanning reads in the range of 65 to 128. The sentence about the bi-modal distribution was removed based on another reviewer’s feedback.

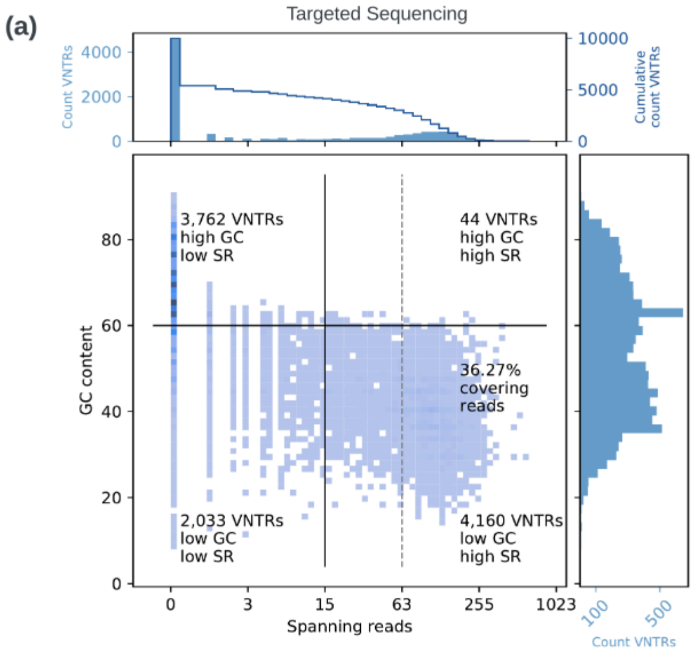

- Figure 2a as appears in the manuscript

4/89
The variance in the number of spanning reads across samples was < 0.2 in a majority (35%) of G-VNTRs, after projecting to maximum value 30, the variance of the spanning reads across samples was < 0.2 for an increased number (68%) of G-VNTRs.
>>35% is not a majority

- Fixed.

>>Why was this "projecting to maximum value 30" done? It seems obvious that if the top number is reduced, the variance would be reduced because you're discarding the variance caused by higher numbers.

Response. While the reviewer has a good point that projecting to the maximum value of 30 reduces the variance, we would like to add that we wanted to measure the variance of the spanning reads in a context that would affect genotyping. With targeted sequencing with PCR, we expect to see an amplified number of reads for VNTRs and it is not surprising to see a large variation of spanning reads across VNTRs. Instead, we wanted to measure how much the variation could affect genotyping.

For any number of spanning reads above 30, the effect on genotyping is minimal or nonexistent. Therefore, we considered any number of spanning reads larger than 30 to be “sufficient”. This way, the VNTRs with more than 30 spanning reads would not skew the variation. This is so that

the variation could in fact measure how many VNTRs we can or cannot genotype with confidence (with 30 spanning reads marking our highest confidence).

4/97
In contrast, 99% (4,160/4,204) of the well-covered VNTRs were located in regions with GC-content < 60%.
>>how is "well-covered" defined?

Response. We define a VNTR “well-covered” if we observe a median of at least 15 spanning reads among samples for that VNTR. This is clarified in the manuscript:

- In contrast, 99% (4,160/4,204) of the well-covered VNTRs **(median of at least 15 spanning reads across samples)** were located in regions with GC-content <60%.

Also, in response to another reviewer’s question, we clarified the use of 15 as the cutoff, by adding the following to Methods.

- To compute a threshold for spanning reads, we estimated the probability of incorrectly calling a heterozygous genotype as homozygous, given *n* spanning reads. Such an incorrect call in AdVNTR occurs when there are 2 or fewer spanning reads from one allele. Considering a case with *n* total reads, *x* reads sampled from one allele, and no allele dropout

$$P_{\text{err}}(n) = P(x \geq n-2 \text{ OR } x \leq 2) = \sum_{i=n-2}^{n} \binom{n}{i} \cdot 0.5^i \cdot 0.5^{n-i} + \sum_{i=0}^{2} \binom{n}{i} \cdot 0.5^i \cdot 0.5^{n-i} \qquad (1)$$

From Eqn. 1, P_err(14) = 0.013, P_err(15) = 0.0074. Thus, n = 15 is the lowest number of reads where the probability of miscalling a heterozygous genotype is lower than 1%, and it was chosen as the threshold for spanning reads.

6/178
We further classified the genomic locations of the P-VNTRs (Figure 3a). They were spread across exons, introns, promoters, 3’ or 5’ untranslated regions.
>>Is there a table of these classifications?

Response. That is a very good point. For each P-VNTR we provide the genomic region in the supplementary table S2. We added a reference to this table, the line highlighted by the reviewer for clarity:

- They were spread across exons, introns, promoters, 3' or 5' untranslated regions **(See Supplemental Table S2)**

7/228
Our results suggest that a large fraction (nearly 60%) of G-VNTRs could not be effectively targeted and did not yield any product.
>>At 4/86, you stated that (32%) of G-VNTRs were not spanned by any read in any sample. Does the 60% include those with less than 15 spanning reads?

Response. That is a very good point. We clarified the statement in the manuscript. 60% includes VNTRs with less than 15 spanning reads:

- Our results suggest that a large fraction (nearly 60%) of G-VNTRs could not be effectively targeted and did not yield **15 or larger spanning reads**.

--- Figures ---
Figure 1
Captions b) c) use of word "Complexity"
>>please use:
"b) Fraction of VNTRs exceeding designated array length"
"c) Fraction of VNTRs exceeding designated motif length"

- Changes applied

Figure 2
What type of scale is used for the coloring in parts (c) and (d)? It's not linear and does not appear to be logarithmic.

- This is a very good point. We have updated the colorbar ticks on figure 2c and d:

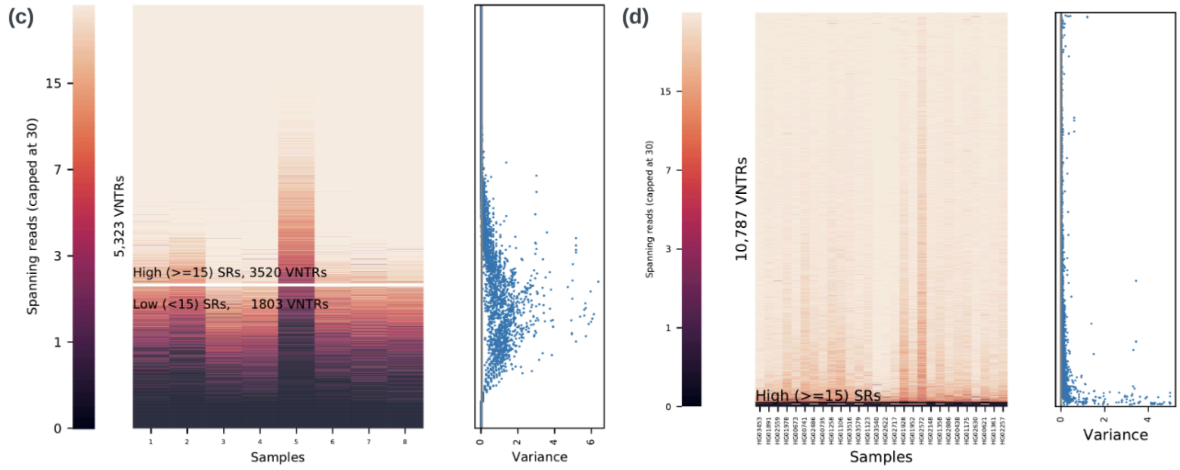

- Updated figure 2c and d

S2

The hue shows the GC content percentile for VNTR and 100bp flanking regions.

>>why are you showing the GC content only around the VNTR region? Are other references to GC content in the manuscript to this narrow region or to the entire read length?

Response. This is a good point. GC-content is computed for the entire VNTR plus 100 bp on each flanking region. Our criteria for GC-content includes the VNTR region as well as the 200 bp in total in the flanking region and is the same definition wherever referenced in the manuscript.

We tested the correlation between the GC-content computed based on a varying threshold on the flanking region size. For example, we compared the GC-content of all the target VNTRs with a 100 bp flanking threshold against 200 bp, 500 bp and 1000 bp flanking region size (all thresholds on each flanking side) using Pearson's correlation and found 0.97, 0.92 and 0.92 correlation respectively. We plotted the GC-content with two thresholds of 100 bp and 1000 bp in Figure R5, included here, but not added to the manuscript.

To pick a threshold on the flanking region size, we considered a value that is relevant in both HiFi and Illumina sequencing. Even though our focus is on HiFi reads in this study, we wanted our VNTR database to be relevant for scientists working on Illumina reads as well. For short Illumina reads, a longer flanking region is not relevant as the reads spanning the VNTR will likely not expand beyond 100 bp on each of the flanking sides.

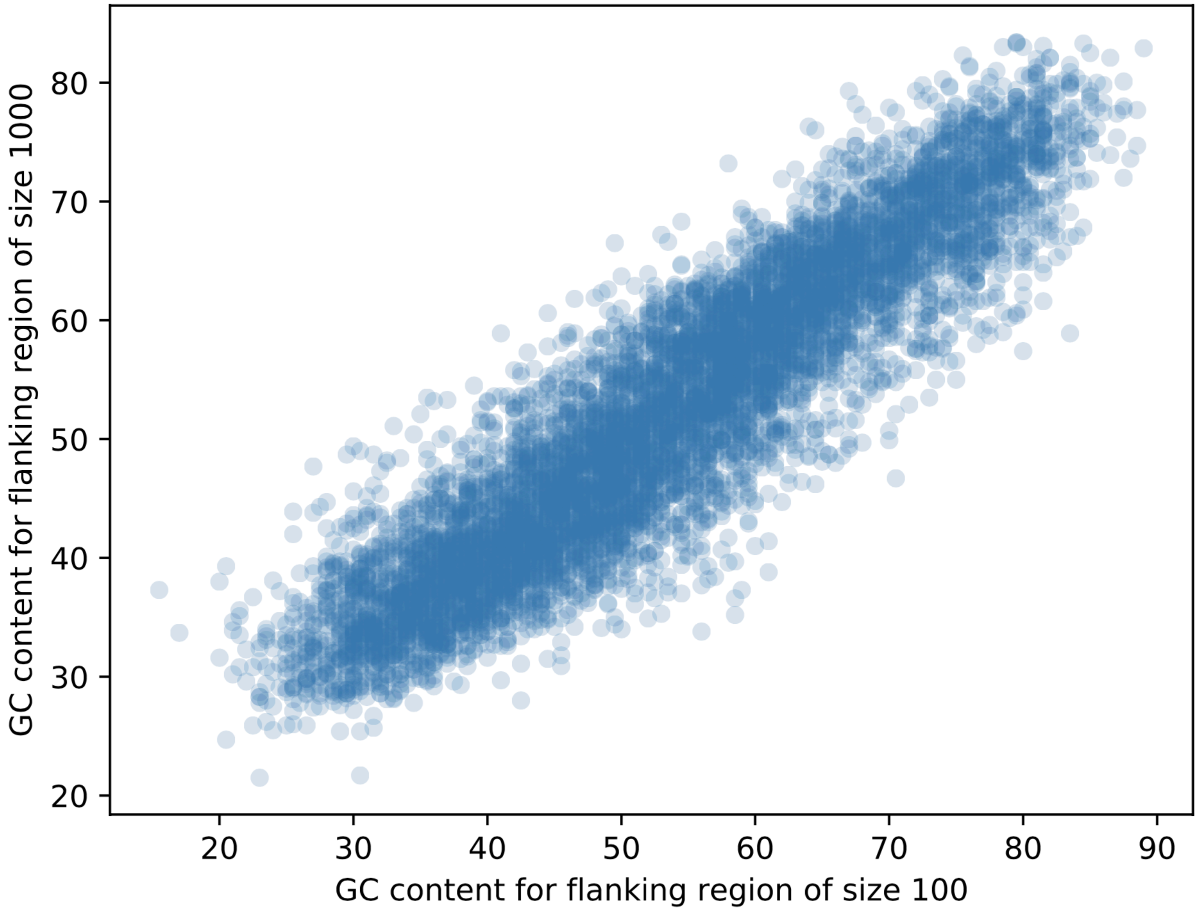

- Figure R5. GC-content of all the target VNTRs computed for the VNTR boundaries as well as a 100 bp on flanking region compared to a threshold of 1000 bp on flanking region (each side).

--- References ---
Not all journal titles are correctly italicized.

Response. According to the submission guidelines on PLOS (https://journals.plos.org/plosone/s/submission-guidelines#loc-style-and-format), the references should be in the "Vancouver" style with some examples provided (https://journals.plos.org/plosone/s/submission-guidelines#loc-references). Within the provided examples, journal titles are not italicized. Please let us know the most updated guidelines (if different from the links we shared above) or confirm that the "Vancouver" style should be used for references.

Reviewer #4 - 0/7 comments left

In this study, the authors present results from comprehensive genotyping of gene-proximal and phenotype-associated VNTRs using targeted Illumina and PacBio HiFi sequencing, and they compare these results with whole-genome PacBio HiFi data. The authors demonstrate good coverage of most VNTR loci of interest (except for GC-rich VNTRs) in their targeted analysis, and report strong Mendelian inheritance consistency between Illumina and PacBio HiFi data. The manuscript is well-written, and the data is presented concisely and neatly. Given the interest in understanding the roles of VNTRs in disease, this paper could make a valuable contribution to the field. For genotyping, the authors used adVNTR, a tool developed by their group, justifying this choice by stating that adVNTR is compatible with both Illumina and HiFi datasets. The reviewer has a few questions and suggestions for the authors to consider, along with minor comments listed below.

1. Please italicize CACNA1C on page 6. There are instances where the gene name is italicized and others where it is not. Consistency would improve readability.

Response. Thanks for pointing it out. We applied the requested changes.

2. There is more data available in the HPRC. Could the authors clarify why they limited their study to 28 individuals?

Response. Even though more than 28 samples are included in the HPRC reference dataset, when we were looking for samples for which whole genome HiFi reads are available, only 28 samples came up at the time of study. Other samples had Illumina reads, but no HiFi reads were provided for them at that time.

3. Although the authors mention the reference sequence used, the version is not specified when first mentioned in line 68. Could they please add the reference genome version?

Response Thanks for pointing it out. We applied the requested changes:

- In total, the **GRCh38** reference DNA sequence of 46% of G-VNTRs and 71% of P-VNTRs exceeded 150 bp

4. While the authors explain why HiFi sequencing data is preferable over ONT, it would be helpful to include a comparison of genotype consistency among Illumina, ONT, and PacBio HiFi. ONT's adaptive sampling feature eliminates the need for PCR, reducing PCR-associated biases (such as low coverage in GC-rich regions) and additional enrichment strategies, while also providing methylation information. ONT has made substantial advancements, and datasets based on ONT could potentially support VNTR genotyping, if not motif composition analysis.

Response. We wanted to limit this paper to a comparison of targeted and whole genome sequencing and wanted to use the same sequencing platform for both to have a more direct comparison. The targeted sequencing is already performed and samples are not available for repeating the experiment with ONT. However, the reviewer's point is excellent. In the discussion we cited the "Miller *et al*" paper and highlighted that sequencing data based on ONT could potentially support VNTR genotyping:

- Long-read sequencing technologies have seen rapid adoption in recent years, leading to an increase in publicly available data that can enhance benchmarking studies. For example, targeted long read sequencing using Oxford Nanopore Technology (ONT) adaptive sampling is another alternative to HiFi reads for genotyping VNTRs. With the advances in ONT adaptive sampling, the target regions can be selected without the need for PCR amplification. Therefore, it is possible to have higher coverage in the high-GC regions where we observed the least number of reads.

5. In line with the last comment, did the authors observe consistency in motif composition for VNTRs spanned by reads in the targeted Illumina and HiFi sequencing data?

Response This is an excellent point. Motif composition analysis can be extremely complex for VNTRs. Most VNTR genotyping tools focus on either estimating the repeat counts [3,4] or reporting the allele sequencing (without breaking it down into motifs) [5,6]. Motif composition analysis requires a complex model to encode motifs as well as the position or order where each motif is located. An example tool for visualizing motif compositions has been developed by our co-author Jonghun Park (Park 2023*). Even though the visualization is very helpful in understanding the composition of a single VNTR in a single sample, given all the reads, further analysis requires a more complex model to systematically analyze the motif composition of VNTRs in a cohort of individuals. Therefore, we believe that motif composition analysis is beyond the scope of the current paper.

- * References: Park J, Kaufman E, Valdmanis PN, Bafna V. TRviz: a Python library for decomposing and visualizing tandem repeat sequences. Bioinformatics advances. 2023 Jan 1;3(1):vbad058.

6. In the trio analysis, where the authors discuss Mendelian consistency, it would be interesting to know if they observed any de novo changes in VNTR motif compositions within the trios.

Response. The reviewer is asking for motif composition analysis on the trios analyzed in this study. For the reasons explained in the previous comment (comment #5) we believe that this is best left for a future study due to the complexities of motif composition analysis.

7. In the abstract, the authors mention that "Illumina reads span only 46% of the 10,787

(G-)VNTRs and 71% of 48 phenotype-associated (P-)VNTRs." It's unclear if these are findings from the current study or general knowledge. If these are study results, the reviewer suggests moving this statement to the last paragraph where the findings are discussed. Otherwise, consider rephrasing to clarify that this is known information.

Response. This is a very good point. We clarified that these VNTRs are findings from this particular study. However, We would like to quantitatively emphasize the importance of HiFi reads in the first paragraph to set the motivation throughout the rest of the abstract. Moving this to the last paragraph would leave a void in our motivation and focus on the HiFi reads for the entire abstract. That is why we believe it is best to keep the statement at the first paragraph of the abstract. However, we have changed it to the following:

- We curated a set of 10,787 gene-proximal (G-)VNTRs, and 48 phenotype-associated (P-)VNTRs of interest for this study. Illumina reads only spanned 46% of the G-VNTRs, and 71% of P-VNTRs, motivating the use of HiFI sequencing.

Have the authors made all data and (if applicable) computational code underlying the findings in their manuscript fully available?
The PLOS Data policy requires authors to make all data and code underlying the findings described in their manuscript fully available without restriction, with rare exception (please refer to the Data Availability Statement in the manuscript PDF file). The data and code should be provided as part of the manuscript or its supporting information, or deposited to a public repository. For example, in addition to summary statistics, the data points behind means, medians and variance measures should be available. If there are restrictions on publicly sharing data or code —e.g. participant privacy or use of data from a third party—those must be specified.

Reviewer #1: **No:** I could not find a data availability statement for the targeted sequencing

Reviewer #2: **No:** Data availability statement missing. Code present.

Reviewer #3: Yes

Reviewer #4: Yes
